# Is *FAM19A5* an adipokine? Peripheral *FAM19A5* in wild-type, *FAM19A5* knockout, and *LacZ* knockin mice

**DOI:** 10.1016/j.mocell.2024.100125

**Published:** 2024-10-18

**Authors:** Hoyun Kwak, Eun-Ho Cho, Eun Bee Cho, Yoo-Na Lee, Anu Shahapal, Hyo Jeong Yong, Arfaxad Reyes-Alcaraz, Yongwoo Jeong, Yerim Lee, Minhyeok Lee, Nui Ha, Sitaek Oh, Jae Keun Lee, Won Suk Lee, Won Kyum Kim, Sangjin Yoo, Soon-Gu Kwon, Jong-Ik Hwang, Jae Young Seong

**Affiliations:** 1 Neuracle Science Co., Ltd., Seoul 02841, Republic of Korea; 2 Department of Biomedical Sciences, Graduate School of Medicine, Korea University, Seoul 02841, Republic of Korea; 3 College of Pharmacy, University of Houston, Houston, TX 77204, USA

**Keywords:** Adipose tissue, Brain, *FAM19A5*, Peripheral expression, S1PR2

## Abstract

*FAM19A5* is a novel secretory protein expressed primarily in the brain. However, a recent study reported that *FAM19A5* is an adipocyte-derived adipokine that regulates vascular smooth muscle function through sphingosine-1-phosphate receptor 2 (S1PR2). In our study, we investigated *FAM19A5* transcript and protein levels in peripheral tissues, including adipose tissues, from wild-type, *FAM19A5* knockout, and *FAM19A5*-*LacZ* knockin mice. We found that the *FAM19A5* transcript levels in the central nervous system were much greater than those in any of the peripheral tissues, including adipose tissues. Furthermore, the *FAM19A5* protein levels in adipose and reproductive tissues were below detectable limits for Western blot analysis and enzyme-linked immunosorbent assay (ELISA). Additionally, we found that the *FAM19A5* protein did not interact with S1PR2 in terms of G-protein-mediated signal transduction, β-arrestin recruitment, or ligand-mediated internalization. Taken together, our findings revealed basal levels of *FAM19A5* transcripts and proteins in peripheral tissues, confirming its primary expression in the central nervous system and lack of significant interaction with S1PR2.

## INTRODUCTION

*FAM19A5* is a member of the FAM19A protein family (also called the TAFA family) and is highly expressed in the brain (Tom Tang et al., 2004). The *FAM19A5* gene encodes a secretory protein with a high degree of amino acid sequence identity across vertebrate species (Jeong et al., 2021), suggesting its functional relevance. Transcripts for *FAM19A5* are abundant in the brain and relatively rare in peripheral tissues (Tom Tang et al., 2004). A recent study using *FAM19A5-LacZ* knockin (KI) mice revealed the expression patterns of *FAM19A5* during embryogenesis and in the adult brain (Shahapal et al., 2019). *FAM19A5* is expressed in the ventricular zone and ganglionic eminence during early brain development, suggesting a role in the proliferation and differentiation of neural stem cells and oligodendrocyte precursor cells (OPCs) (Shahapal et al., 2019). In the adult brain, X-gal staining combined with immunostaining for cell-type markers revealed that *FAM19A5* is expressed in diverse cell types, including neurons, astrocytes, and OPCs (Shahapal et al., 2019). These findings are consistent with single-cell RNA sequencing data from mouse and human brains (Karlsson et al., 2021, Mathys et al., 2019, Sjöstedt et al., 2020, Zeisel et al., 2018).

The results from the *FAM19A5* knockout (KO) and *FAM19A5*-*LacZ* KI mouse models suggest that *FAM19A5* may affect the formation and elimination of synapses in neurons (Huang et al., 2021, Shahapal et al., 2024). Furthermore, we recently demonstrated that *FAM19A5* contributes to synapse elimination by binding to leucine rich repeat containing 4B (LRRC4B), a postsynaptic cell adhesion molecule, through the *FAM19A5*-binding domain within LRRC4B (Kim et al., 2024). LRRC4B is known to participate in synapse formation by binding to protein tyrosine phosphatase receptor type F (PTPRF), a cell adhesion molecule in the presynapse (Woo et al., 2009). However, when *FAM19A5* binds to LRRC4B, the interaction between LRRC4B and PTPRF is inhibited. Conversely, treatment with an anti-*FAM19A5* antibody that inhibits the function of *FAM19A5* restored the lost synapses in the hippocampal neurons. This action of the FAM19A5 antibody also restored the loss of synapses and cognitive function in mouse models of Alzheimer's disease (Kim et al., 2024, Park et al., 2024).

In addition to its functional importance in the brain, a recent study showed that *FAM19A5* suppressed neointima formation in injured rat carotid arteries by interacting with sphingosine-1-phosphate receptor 2 (S1PR2) (Wang et al., 2018). This study indicated that *FAM19A5* is produced primarily in adipose tissues. Quantitative real-time polymerase chain reaction (qRT-PCR) analysis of human tissue samples revealed that the *FAM19A5* mRNA level in adipose tissue was greater than that in brain tissue. Furthermore, immunohistochemistry and ELISA for *FAM19A5* have shown high levels of the *FAM19A5* protein in human and rodent adipose tissues (Wang et al., 2018). Additionally, *FAM19A5* mRNA and protein levels are significantly downregulated in obese and diabetic animals (Wang et al., 2018), suggesting a causal relationship between decreased *FAM19A5* levels and metabolic disease conditions. However, this result partially contradicts recent public database reports on human tissue RNA expression, which show that *FAM19A5* is expressed in female and male reproductive tissues, including the ovaries, uterus, and testis, although its absolute levels in these tissues are significantly lower than those in brain tissue (Karlsson et al., 2021, Sjöstedt et al., 2020).

In the present study, we examined the basal levels of *FAM19A5* transcripts and proteins in a variety of peripheral tissues via qRT-PCR, Western blotting, and ELISA. The methods were verified with *FAM19A5* KO mice. We also identified the cell types that express *FAM19A5* via X-gal staining of peripheral tissues from *FAM19A5-LacZ* KI mice, as previously described (Shahapal et al., 2019). In contrast to a previous report (Wang et al., 2018), this study also demonstrated that the *FAM19A5* transcript and protein levels in adipose tissues are very low compared with those in brain tissues and that S1PR2 does not interact with the *FAM19A5* protein, as determined by receptor-mediated signal transduction, β-arrestin recruitment, ligand-mediated receptor internalization, and coimmunoprecipitation (Co-IP).

## MATERIALS AND METHODS

### Animals

C57BL/6J mice were purchased from Nara Biotech or Orient Bio, Inc. Male db/db mice were purchased from Central Lab Animal Inc. The mice were housed under temperature-controlled (22-23°C) conditions with a 12-hour light/12-hour dark cycle. The mice were given standard chow and water ad libitum. All the animal experiments were designed to use the fewest mice possible, and anesthesia was administered when necessary. All animal procedures were approved by the Institutional Animal Care and Use Committee of Korea University (KOREA-2019-0076 and KOREA-2019-0032).

### Generation of *FAM19A5* KO Mice

*FAM19A5* KO mice were generated by ToolGen, Inc using the clustered regularly interspaced short palindromic repeat/CRISPR-associated protein 9 (CRISPR/Cas9) system (Lee et al., 2018). Briefly, single-guide RNAs (sgRNAs) were designed to target a 5′-UTR sequence of exon 1 (left sgRNA, ccgtctctgtcgccatccagagg E1-1) and the 3′-UTR sequence of exon 4 (right sgRNA, cttggcacttaactcccagatgg). Cas9 mRNA and sgRNAs were microinjected into the cytoplasm of C57BL/6J mouse zygotes, and the resulting embryos were transferred into the oviducts of IcrTac:ICR pseudopregnant foster mothers to produce live founder mice. Founder mice with mutant alleles were screened via genomic DNA PCR to amplify the genomic region spanning the sgRNA target sites. The primer sequences for the founder screening PCR were as follows: forward, GGGGGTCCCAAGTCACCTAAC; reverse, AAGAACTTGGGAGACAGGCAAA. The PCR products from the founder mice were cloned, and the corresponding mutations were identified by direct-sequencing analysis (Bionics Co, Ltd). Founder mice with a mutant allele that lacked the 125,000-bp sequence containing exons 1 to 4 were bred with wild-type (WT) mice. Germline transmission of the mutant allele was determined by genotyping with the following primers: gF1, TCGGTTCACTTTCCGGATCAAT; gR1, AAGAACTTGGGAGACAGGCAAA; and gF2, TCCTGGGAGAGGGGAATAGTTT. Homozygous *FAM19A5* KO mice were generated by heterozygous intercross breeding.

### *FAM19A5-LacZ* KI Mice

*FAM19A5-LacZ* KI mice were generated by the UC Davis Mouse Biology Program as previously described (Shahapal et al., 2019). The gene-trap method using *LacZ* as a reporter gene was employed to visualize *FAM19A5* expression in tissue sections. Briefly, the target vector containing the *internal ribosome entry site* (*IRES)-lacZ* gene was inserted in front of exon 4 of the *FAM19A5* gene. The *LacZ* gene is expressed independently of the target *FAM19A5* gene due to an IRES element. This *FAM19A5*-targeting vector was delivered to embryonic stem cells by electroporation. The incorporation of the vector into the target chromosome was confirmed by genotyping and chromosome counting of transgenic embryonic stem cells. The selected transgenic embryonic stem cells were injected into blastocysts, and the embryos were implanted into the uterus of female recipient mice. A germline transmission test was performed to check for stable germline expression in the chimeric generation. The generated *FAM19A5-LacZ* KI chimeric mice were backcrossed onto a C57BL/6J genetic background.

### Quantitative Real-Time Polymerase Chain Reaction Analysis

TRIzol reagent (Invitrogen) was used to isolate total RNA from mouse tissues. The analyzed tissues included the cerebral cortex, hippocampus, spinal cord, heart, aorta, kidney, lung, stomach, small intestine, large intestine, pancreas, liver, salivary gland, thymus, spleen, bone marrow, adrenal gland, pituitary gland, thyroid gland, skeletal muscle, white adipose tissue, brown adipose tissue, and skin. One microgram of RNA was reverse-transcribed into complementary DNA (cDNA) with a RevertAid First Strand cDNA Synthesis Kit (Thermo Scientific). The sequences of the primers used for qRT-PCR were as follows: mFAM19A5-Iso1-F, GTCCTCAACTTTTTGGGCATTC; mFAM19A5-Iso2-F, GCGATGCAGCTCCTGAAG; mFAM19A5-Iso1,2-R, CCCGGTCTAGGGTCACAA; mFAM19A5-Total-F, GGCAGATAGCAGGCACCACT; mFAM19A5-Total-R, GCTGCGATTGTCAGGAGACC; mGAPDH-F, ATCCTG CACCACCAACTGCT; and mGAPDH-R, GGGCCATCCACAGTCTTCTG. The CFX96 Touch RT-PCR detection system with SsoAdvanced Universal SYBR Green Supermix (Bio-Rad) was used for qRT-PCR. Gene expression was normalized to glyceraldehyde 3-phosphate dehydrogenase (GAPDH) levels, and the relative quantity of mRNA was calculated using the comparative Cq method. Possible genomic DNA contamination for all tissues was assessed by performing identical PCRs on samples that had not been reverse-transcribed and showed either no contamination or negligible levels of contamination that did not interfere with qRT-PCR.

### X-Gal Staining

X-gal staining was performed as previously described (Shahapal et al., 2019) using 10-week-old adult WT littermates (2 males) and *FAM19A5-LacZ* KI heterozygote (1 male) and homozygote (3 males and 1 female) mice. The mice were perfused with normal saline and 4% paraformaldehyde in 1× phosphate-buffered saline (PBS). Subsequently, the brain and peripheral tissues were isolated, fixed in 4% paraformaldehyde for 3 h at 4°C, and cryoprotected with 30% sucrose in 1× PBS for 36 h at 4°C. The cryoprotected tissues were embedded in optimal compound temperature solution and 30% sucrose (2:3 ratio). The embedded blocks were sectioned at 20 µm using a cryostat (Leica). Cryosections that were mounted on glass slides were washed twice with 1× PBS for 5 min each, permeabilized with 0.01% sodium deoxycholate and 0.02% Igepal CA-630 in 1× PBS for 15 min, and incubated in X-gal solution for 24 h at 37°C in the dark. The X-gal solution contained 1 mg/ml X-gal, 2 mM magnesium chloride, 5 mM ethylene glycol-bis(2-aminoethylether)-N,N,N′,N′-tetraacetic acid, 5 mM potassium ferrocyanide, 5 mM potassium ferricyanide, 0.01% sodium deoxycholate, and 0.02% Igepal CA-630 in 0.1 M phosphate buffer at pH 7.4. X-gal-stained sections were washed twice with 1× PBS for 5 min each, counterstained with Mayer’s hematoxylin, dehydrated with graded ethanol and xylene, and mounted with a cover glass.

The tissue-specific X-gal signal was scored according to the following criterion: no X-gal signal in either WT or *FAM19A5-LacZ* KI mice was indicated by “-”. The nonspecific X-gal signal detected in both the WT and *FAM19A5-LacZ* KI mice, which was likely due to endogenous β-gal, was represented by “Δ”. An ambiguous signal in both WT and *FAM19A5-LacZ* KI mice was indicated by “Δ/+”. Ambiguity was defined as a signal in *FAM19A5-LacZ* KI mice that appeared stronger than the signal in WT mice or a negligible signal that was found only in *FAM19A5-LacZ* KI mice. A positive signal observed only in *FAM19A5-LacZ* KI mice but not in WT mice is denoted by “+”. Among the samples with a positive signal, “1+” indicated a faint positive signal with a sparse, cellular blue precipitate, “2+” indicated a weak positive signal with one or more blue precipitate areas per cell, “3+” indicated a moderate positive signal that was observed in many cells, and “>4+” indicated a robust positive signal.

### Purification and Fluorescence Labeling of the *FAM19A5* Protein

Recombinant N-terminal His-tagged *FAM19A5* (N-HIS-FAM19A5) with a tobacco etch virus protease recognition sequence was cloned and inserted into the vector pCAG1.1 and expressed in Expi293F cells (Gibco). The supernatant of the transfected cells encoding N-HIS-FAM19A5 was collected for Ni-nitrilotriacetic acid (Ni-NTA) affinity chromatography (Cytiva). Purified N-HIS-FAM19A5 was then digested overnight at 30°C with AcTEV protease (Invitrogen) to remove the His tag. Following the cleavage reaction, the digestion products were verified by sodium dodecyl sulfate–polyacrylamide gel electrophoresis (SDS-PAGE) and Western blot analysis. Purified *FAM19A5* was used as a Western blotting control.

### N-Terminal Sequencing

The purified *FAM19A5* protein was resolved by SDS-PAGE and transferred to a hydrophobic polyvinylidene fluoride membrane (Whatman). The membrane was then stained with Coomassie blue staining solution. The stained *FAM19A5* band was sequenced by the eMass Analysis Lab. N-terminal sequence analysis was performed with a protein sequencer (PPSQ-53A, Shimadzu co) via the Edman degradation method.

### Generation of Anti-*FAM19A5* Antibodies

To generate anti-*FAM19A5* chimeric human/chicken monoclonal antibodies, chickens (*Gallus gallus domesticus*) were immunized with purified N-terminal His-tagged *FAM19A5* protein. Total RNA was extracted from the spleen, bursa of Fabricius, and bone marrow of the immunized chickens, and cDNA was synthesized. From the synthesized cDNA, a single-chain variable fragment (scFv) library was constructed with the pComb3X-SS vector system (Scripps Research Institute). The helper phage VCM13 was added to the culture media for the extraction of phagemid DNA and phage-displayed scFv. Biopanning was performed using recombinant N-HIS-FAM19A5-coated magnetic beads with the phage-displayed scFv. Phages bound by biopanning were amplified and used for a second round of biopanning. Biopanning was repeated 5 times, and each time, the number of washes was increased to identify the phage with the highest affinity. ELISA was used to test the affinity of randomly selected phage-displayed scFv clones from biopanning for recombinant *FAM19A5*. The human *CК* gene was linked to the anti-*FAM19A5* antibody sequence that encodes the light-chain variable region, and the *CH1*, *CH2*, and *CH3* genes of the human immunoglobulin isotype *IgG1* were added to the heavy-chain variable region (GenScript). Vectors encoding the anti-*FAM19A5*-IgG1 antibody were transfected into HEK293F cells, and the recombinant antibody was purified using Protein A beads (RepliGen). Anti-*FAM19A5* antibodies that recognized the epitopes formed at the N-terminal and C-terminal regions were generated and called N-A5-Ab and C-A5-Ab, respectively. The purified antibodies were conjugated with horseradish peroxidase (HRP) using an antibody labeling kit (Abcam) according to the manufacturer’s instructions.

### Western Blot Analysis

Mouse tissue samples were homogenized in a buffer containing 20 mM Tris-HCl (pH 7.5), 500 mM NaCl, 5 mM MgCl_2_, and a protease inhibitor cocktail (Thermo Scientific). Cell lysates were prepared in lysis buffer containing 20 mM Tris-HCl (pH 7.5), 150 mM NaCl, 0.5% NP-40, and protease inhibitor cocktail. To remove N-glycosylation from recombinant *FAM19A5* isoform 1, isoform 2-expressing cell lysate, and brain lysate, the samples were denatured and deglycosylated with PNGase F enzyme according to the manufacturer’s instructions (New England Biolabs). Protein quantification of the homogenized tissues and cell lysates was performed via the Pierce BCA protein assay (Thermo Scientific). The brain homogenates, cell lysates, and cell culture media were then resolved on Tris-glycine gels (Invitrogen) and transferred to prewetted polyvinylidene difluoride (PVDF) blotting membranes using a Trans-Blot Turbo apparatus (Bio-Rad). The membrane was blocked in Tris-buffered saline containing 0.05% Tween-20 and 5% skim milk, followed by overnight incubation with HRP-conjugated N-A5-Ab at 4°C. The blots were washed 3 times with Tris-buffered saline containing 0.05% Tween 20. After the application of enhanced chemiluminescence (ECL) reagents (Thermo Scientific), the immunoreactive bands were visualized (photographed) using a Mini HD9 (UVerytec) system.

### Coimmunoprecipitation (Co-IP)

HEK293 cells were electroporated with reagents from the Neon Transfection System (Invitrogen) following the manufacturer’s instructions. For Co-IP experiments, the cells were treated with 1 μM *FAM19A5* for 30 min. After being washed twice with ice-cold DPBS (Gibco), the cells were resuspended in lysis buffer containing 20 mM Tris-HCl (pH 7.4), 150 nM NaCl, 0.5% NP-40, and a protease and phosphatase inhibitor cocktail (Thermo Scientific). The supernatants were collected by centrifugation at 14,000 rpm for 15 min at 4°C, then mixed with anti-FLAG M2 affinity gel (Sigma). The mixtures were incubated at 4°C for 3 h on a rotating mixer. The beads were spun down and washed 3 times with a washing buffer containing 20 mM Tris-HCl (pH 7.4), 500 mM NaCl, and 0.5% NP-40. The immunoprecipitated complexes were dissolved in SDS-PAGE buffer with a reducing agent and boiled. The *LRRC4B*, *S1PR2*, and *neurexin* 1 (*NRXN1)* genes used in the Co-IP experiments were all purchased from GenScript.

### ELISA

To measure *FAM19A5* in biofluids and tissues from WT, *FAM19A5* KO, and db/db mice, 96-well microplates were coated with the 6xHis-TEV-LRRC4B (453-576) protein in carbonate buffer (pH 9.6) and incubated overnight at 4°C. The next day, the plates were washed twice with washing buffer (PBS with 0.05% Tween 20) via a microplate washer (Tecan) and tapped dry. Blocking buffer was then added to each well, and the plates were incubated at 37°C for 1 h. After 2 additional washes, the standard solution and samples were added to each well and incubated at room temperature for 1 h. Following 5 washes, HRP-conjugated C-A5-Ab in blocking buffer was added to each well and incubated at 37°C for 1 h. Then, TMB solution (Thermo Scientific) was added to each well, and the samples were incubated at room temperature for 20 min. The reaction was stopped with sulfuric acid, and the optical density was measured at 450 nm via a microplate reader (Molecular Devices).

### SRE-Luciferase Assay for S1PR2 Activation

The *SRE-luciferase* (*SRE-luc*) vector containing a single copy of the serum response element (SRE: CCATATTAGG) conjugated to luciferase was purchased from Stratagene. The cDNAs for human S1PR2 were obtained from BRN Science, Inc. The cDNA genes were inserted into the *Eco*RI and *Xho*I sites of the pcDNA3.1 vector (Invitrogen). Previously, we constructed a HEK293 cell line that stably expresses Gqi and allows for the induction of Gq-dependent signaling pathways upon activation of a Gi-coupled receptor (Kim et al., 2014). For the luciferase assays, HEK293Gqi cells were seeded on 48-well plates. Transfections were performed using a mixture of the SRE-luc reporter construct, S1PR2 expression plasmid, and Lipofectamine 2000 (Invitrogen), and the cells were incubated in Opti-MEM (Gibco) for 20 min before being applied to the cells. After transfection, the cells were incubated in serum-free Dulbecco's modified eagle medium (DMEM) for 16 to 18 h and then treated with either sphingosine-1-phosphate (S1P, Sigma) or purified *FAM19A5* protein for 6 h. The cells were lysed by adding lysis buffer to the wells. Luciferase activity was determined by analyzing each cell extract in a luciferase assay system according to the standard protocol for the Synergy 2 Multi-Mode Microplate Reader (BioTek).

### Confocal Imaging of S1PR2 and *FAM19A5* Internalization

HEK293 cells were seeded at a density of 4.5 × 10^4^ cells/well on poly-L-lysine-coated plastic film coverslips in 12-well plates. The following day, the cells were transfected with *S1PR2-GFP* plasmids. One day post transfection, the cells were incubated in serum-free MEM for 16 h prior to treatment with S1P (Sigma) and *FAM19A5*. Images of the cells were taken 30 min after ligand treatment via a confocal laser scanning microscope (Leica TCS-SP8).

### NanoLuc Luciferase Complementation Assay

The NanoLuc luciferase complementation assay to detect S1PR2 and β-arrestin interactions was performed as previously described (Reyes-Alcaraz et al., 2018, Reyes-Alcaraz et al., 2019). Briefly, the NanoBit starter kit containing the plasmids and the necessary reagents to complete the structural complementation assays in this study was a gift from the Promega Company. HEK293 cells were maintained in DMEM supplemented with 10% fetal bovine serum (FBS) and penicillin-streptomycin (Gibco). One day prior to transfection, the cells were seeded in poly-L-lysine-coated 96-well plates at a density of 2.5 × 10^4^ cells per well. A mixture containing 100 ng of β-arrestin construct with SmBit, 100 ng of S1PR2 with LgBit or Nluc, and 0.4 μl of Lipofectamine 2000 was prepared and added to each well. Six hours post transfection, the medium was aspirated and replaced with serum-free DMEM. Twenty-four hours post transfection, the medium was aspirated and replaced with 100 μl of Opti-MEM at room temperature. After a 10-minute incubation at room temperature, 25 μl of furimazine substrate was added, and luminescence measurements were recorded every minute for 10 min. Next, 10 μl of ligand was added to each well, and luminescence measurements were recorded immediately for 30 min at 1-minute intervals (Synergy H1 Hybrid Multi-Mode Reader, BioTek).

### Statistical Analysis

All the statistical analyses were conducted via GraphPad Prism 8 software (GraphPad Software, Inc). The data are presented as the means ± standard errors of the means. For multiple comparisons, one-way analysis of variance (ANOVA) followed by Tukey’s post hoc corrections was employed. A *P*-value less than .05 was considered statistically significant.

## RESULTS

### *FAM19A5* mRNA Levels in Peripheral and Brain Tissues

**Fig. 1:**
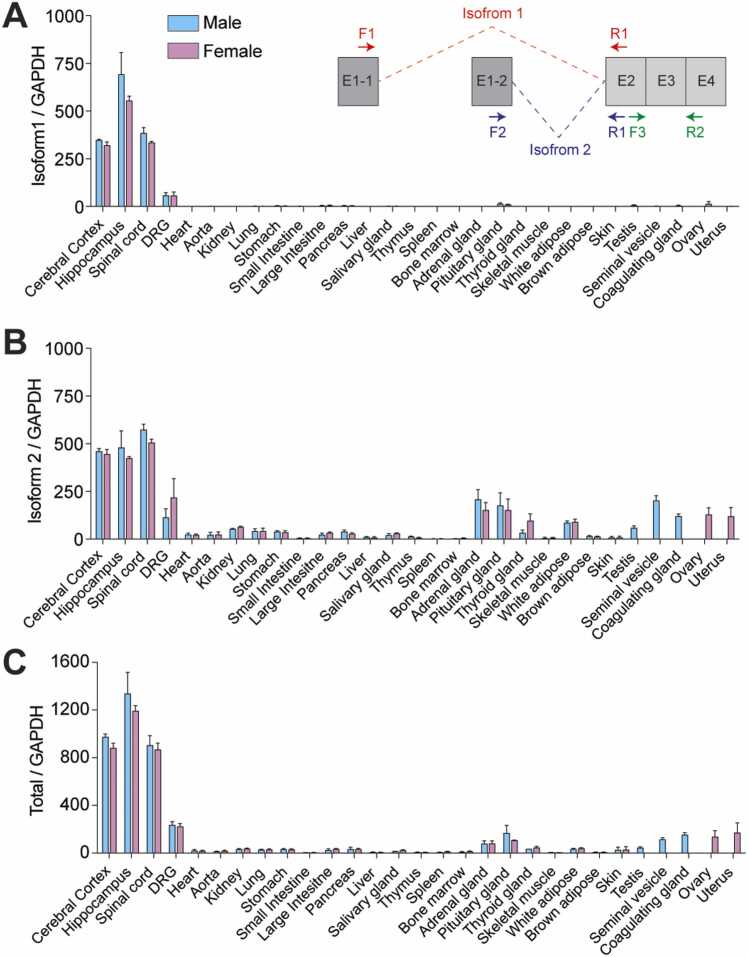
*FAM19A5* transcript levels in central and peripheral tissues. qRT-PCR analysis of *FAM19A5* (A) isoform 1 and (B) isoform 2, and (C) total transcript levels in the indicated tissues. (insert in A) A schematic diagram illustrating the PCR primer locations used to determine the *FAM19A5* isoform 1, isoform 2, and total *FAM19A5* transcripts. Isoform 1 and isoform 2 differ in their exon 1 sequences (E1-1 for isoform 1 and E1-2 for isoform 2) and share exons 2 to 4. The dashed lines indicate splicing junctions for isoform 1 (red) and isoform 2 (blue). Isoforms 1 and 2 and total transcript levels were analyzed using the F1-R1, F2-R2, and F3-R3 primers, respectively. The values are presented as the means ± standard errors of the means from 3 independent experiments.

The human and mouse *FAM19A5* genes can produce 2 transcript isoforms, isoform 1 and isoform 2, due to the alternative use of the first exon, exon 1-1 and exon 1-2 (Wang et al., 2018). To determine the *FAM19A5* transcript levels, tissue samples from the central nervous system (CNS) and peripheral nervous system (PNS) were analyzed via qRT-PCR. Additionally, tissue samples from other peripheral tissues were also analyzed by qRT-PCR. Transcript levels were detected with specific primer sets for *FAM19A5* isoform 1, *FAM19A5* isoform 2, and the total transcript (Fig. 1A, insert). The total *FAM19A5* transcript levels were approximately equal to the sum of the isoform 1 and isoform 2 levels (Fig. 1A-C and Table 1).

**Table 1:** *FAM19A5* expression profile of mouse peripheral tissues using qRT-PCR and X-gal staining of wild-type and *FAM19A5-LacZ* KI (+/+) mice

Organ systems	Peripheral tissues	*FAM19A5/GAPDH*						*LacZ* expression		
		Total		Iso1		Iso2		Male	Female	Cell type
		Male	Female	Male	Female	Male	Female			
CNS	Cortex	976.5	882.9	348.3	321.2	460.8	447.0	>4+	>4+	+: Neuron
	Hippocampus	1339.5	1194.3	694.0	557.1	480.3	425.5	>4+	>4+	
	Spinal cord	906.2	869.4	385.4	335.6	575.7	506.6	>4+	>4+	
PNS	DRG	235.8	224.9	58.9	58.1	114.7	218.7	4+	4+	
Cardiovascular system	Heart	21.9	19.2	0.4	0.3	24.6	22.9	2+	2+	+: Cardiac muscle cell
	Aorta	13.9	18.0	0.6	0.2	22.5	24.5	-	NT	-
Urinary system	Kidney	34.4	38.9	0.1	0.1	54.6	65.1	Δ; Δ/+	Δ; Δ/+	Δ: Glomeruli Δ/+: renal tubular epithelium
Respiratory system	Lung	28.6	30.9	1.3	0.9	43.8	43.8	Δ	Δ	Δ: Alveolar macrophage and bronchiole epithelium
Alimentary system	Stomach	33.3	30.3	3.9	3.1	40.6	36.6	1+; Δ	1+; Δ	+: Smooth muscle cell/myenteric plexusΔ: Mucosal cell
	Small intestine	4.2	3.6	2.3	0.1	5.2	4.2	1+; Δ	1+; Δ	
	Large intestine	26.9	35.4	4.5	6.3	23.6	33.2	1+; Δ	1+; Δ	
	Pancreas	34.7	33.4	4.0	3.5	40.0	29.2	-	-	-
	Liver	8.2	5.7	ND	ND	11.4	8.3	-	-	-
	Salivary gland	14.9	24.5	1.9	2.3	21.8	30.9	Δ	NT	Δ: Mucous and serous acinar cells
Lymphoid system	Thymus	8.0	7.1	0.4	0.3	13.7	8.3	Δ	Δ	Δ: Some cells in both cortex and medulla
	Spleen	7.1	12.7	0.2	0.3	1.5	2.2	Δ	Δ	Δ: Some cells in both red pulp and white pulp
	Bone marrow	10.7	14.7	ND	0.3	2.6	5.4	-	-	-
Endocrine system	Adrenal gland	80.5	81.8	1.7	0.6	208.7	153.0	-	1+	+: X-zone cell
	Pituitary gland	170.2	107.1	14.1	10.8	177.1	152.5	-	NT	-
	Thyroid gland	37.8	46.4	0.8	1.0	33.5	97.2	Δ	NT	Δ: Follicular cell
	Pancreatic islet	NT	NT	NT	NT	NT	NT	-	-	-
Musculoskeletal system	Skeletal muscle	4.3	4.2	0.1	0.1	6.6	6.9	-	-	-
Adipose tissue	White adipose tissue	35.7	41.9	0.6	0.6	87.2	91.9	-	-	-
	Brown adipose tissue	8.2	7.6	0.2	0.2	16.8	13.6	-	-	-
Integumentary system	Skin	28.2	29.5	ND	ND	10.0	10.0	Δ	NT	Δ: Sebocyte
Male reproductive system	Testis	45.1	NA	7.0	NA	60.1	NA	3+; Δ	NA	+: Spermatogenic cells Δ: Leydig cell
	Seminal vesicle	115.3	NA	1.5	NA	203.5	NA	-	NA	-
	Coagulating gland	154.9	NA	5.2	NA	121.6	NA	Δ	NA	-
Female reproductive system	Ovary	NA	137.3	NA	14.3	NA	130.0	NA	1+; Δ	+: Oocyte Δ: Granulosa cell and lutein cell
	Uterus	NA	172.9	NA	1.1	NA	120.5	NA	1+; Δ	+: Smooth muscle cell in myometrium Δ: Epithelium in endometrium

qRT-PCR, quantitative real-time polymerase chain reaction; CNS, central nervous system; PNS, peripheral nervous system; DRG, dorsal root ganglion; ND, not detected; NT, not tested; NA, not applicable. The criteria for X-gal intensity scoring are described in detail in the “Materials and Methods” section. Briefly, Δ and Δ/+ represent nonspecific and ambiguous signals, respectively. The notations 1+, 2+, 3+, and 4+ indicate faint, weak, moderate, and robust positive signals, respectively.

Tissues from the CNS exhibited the highest levels of isoform 1/2 and total *FAM19A5* transcripts. The dorsal root ganglion, a representative PNS tissue, also exhibited high *FAM19A5* transcript levels. In these CNS and PNS tissues, the *FAM19A5* transcript levels were not significantly different between male and female mice (Fig. 1A-C and Table 1).

In general, peripheral tissues exhibited very low levels of *FAM19A5* transcripts, ranging between 1/100 and 1/10 of the total transcript levels found in CNS tissues. Notably, isoform 1 expression was negligibly low in all the examined peripheral tissues. In contrast, isoform 2 was moderately expressed in reproductive tissues and endocrine tissues, including the thyroid, pituitary, and adrenal glands. Other tissues, including white and brown adipose tissue, exhibited low levels of total transcripts and isoform 2 (Fig. 1A-C and Table 1).

### *FAM19A5* Expression in Peripheral Tissues From *FAM19A5*-*LacZ* KI Mice

Previously, we used X-gal staining of tissues from *FAM19A5-LacZ* KI mice to identify the cell types expressing the *FAM19A5* transcript. This staining revealed positive X-gal signals in various cell types, including neurons, astrocytes, and OPCs (Shahapal et al., 2019). This result aligns with the findings from single-cell RNA sequencing data of mouse and human brains available in independent articles and public databases such as mousebrain.org and the Human Protein Atlas (Karlsson et al., 2021, Mathys et al., 2019, Sjöstedt et al., 2020, Zeisel et al., 2018).

In this study, we further identified peripheral tissues that express *FAM19A5* in *FAM19A5-LacZ* KI mice. X-gal staining was performed on various peripheral tissues (Supplementary Table 1 and Supplementary Figs. 1-24). *FAM19A5* promoter-driven X-gal signals were not observed in most tissues. The exceptions included subsets of cardiac muscle cells, smooth muscle cells or mesenteric plexuses of the gastrointestinal tract, inner cortical cells of the X-zone in the adrenal gland, myometrial smooth muscle cells in the uterus, and germ cells in the testis and ovary (Fig. 2 and Supplementary Figs. 1-24). The X-gal signal intensity in these cells was very faint to moderate compared to that in the hippocampus of the brain (Fig. 2). X-gal staining in white and brown adipose tissues was not detected, even though these tissues were reported to express high levels of *FAM19A5* (Wang et al., 2018).

**Fig. 2:**
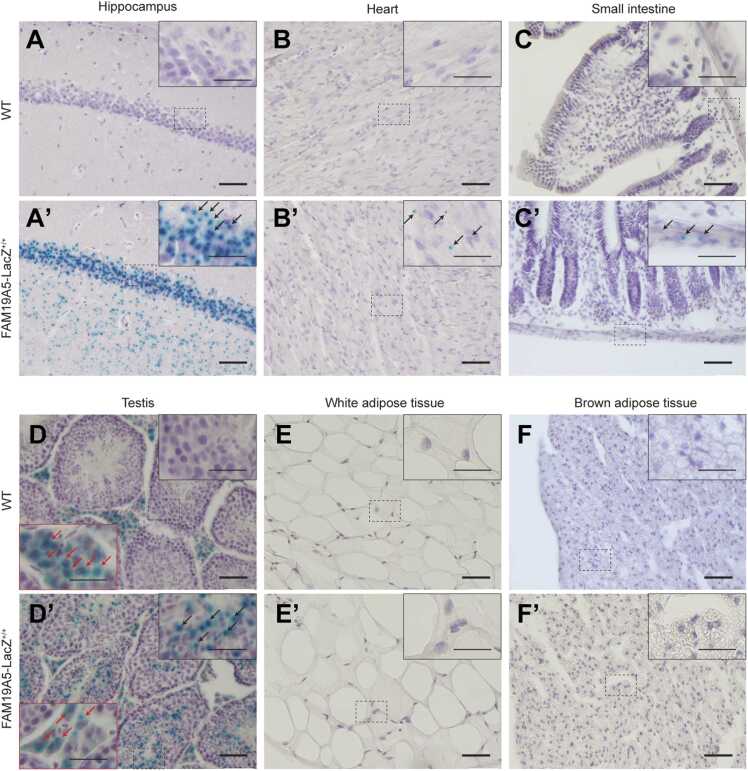
X-gal staining of peripheral tissues from WT and *FAM19A5-LacZ* KI^+/+^ mice. The cryosections were stained with X-gal and counterstained with hematoxylin. Representative light photomicrographs of (A, A′) the brain hippocampus, (B, B′) heart, (C, C′) small intestine, (D, D′) testis, (E, E′) white adipose tissue, and (F, F′) brown adipose tissue from WT (A-F) and *FAM19A5-LacZ*^+/+^ (A′-F′) mice. The images in the dashed boxes are magnified in the upper right. Dot-shaped blue precipitates were present only in the *FAM19A5-LacZ*^+/+^ samples, as indicated by the black arrows (A′, B′ C′, and D′). Dispersed precipitates were observed in both the WT and *FAM19A5-LacZ*^+/+^ samples and are indicated by red arrows (D and D′). The scale bars in the inset represent 20 µm.

The X-gal signals observed in WT mice were likely due to tissue-specific high endogenous β-gal expression (Johnson et al., 1986, Trifonov et al., 2016). The endogenous β-gal-driven X-gal-positive tissues included glomeruli and renal tubules in the kidney; individual cells in the gastrointestinal mucosa; alveolar macrophages; bronchiole epithelia in the lung, thymus, and spleen immune cells; thyroid follicles; sebaceous glands in the skin; and the coagulating gland epithelium, Leydig cells in the testis, endometrial glandular epithelium in the uterus, and granulosa and lutein cells in the ovary (Fig. 2, Supplementary Table 1, and Supplementary Figs. 1-24). Overall, the X-gal signal intensity in peripheral tissues is highly correlated with the *FAM19A5* transcript levels measured by qRT-PCR, as well as with mouse and human data from public databases (Supplementary Table 2).

### *FAM19A5* Proteins in HEK293 Cells Expressing Each Type of *FAM19A5* Isoform

**Fig. 3:**
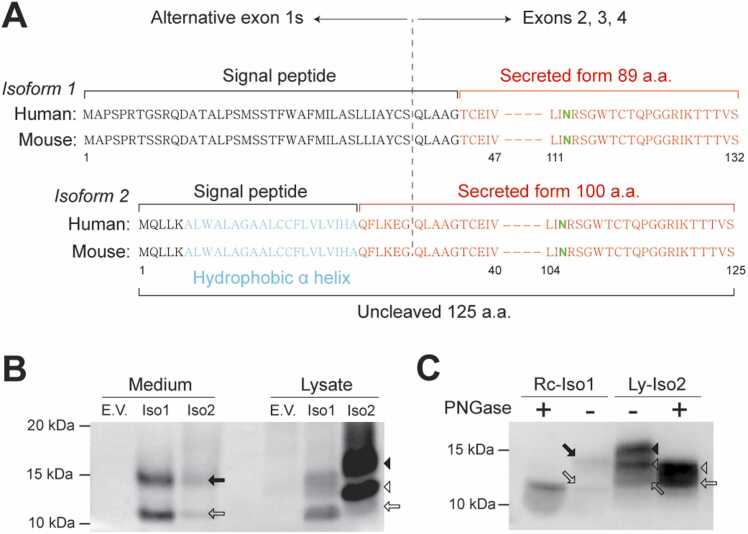
*FAM19A5* isoform 1 and 2 proteins. (A) Diagram illustrating the N-terminal differences between isoform 1 and isoform 2. Human and mouse amino acid sequences for both isoforms are aligned, with residue numbers indicated below the sequences. The N-terminal regions of isoform 1 and isoform 2 differ due to the presence of alternative exon 1s, while the sequences from exon 2 to exon 4 are identical. The boundary between exon 1 and exon 2 is marked by a dashed line. The signal peptide and secreted sequences are represented in black and red, respectively. The hydrophobic α-helix is shown in cyan, and the potential glycosylation site is highlighted in green. (B) Western blot analysis of *FAM19A5* in the culture medium and lysates from HEK293 cells expressing either FAM19A5-Iso1 or FAM19A5-Iso2. Cells transfected with an empty vector (E.V.) served as the control group. (C) Western blot analysis of recombinant isoform 1 (Rc-Iso1) and lysate-derived isoform 2 (Ly-Iso2) *FAM19A5* proteins treated with PNGase F. The open and closed arrows represent nonglycosylated and glycosylated secreted *FAM19A5* proteins, respectively. The open and closed arrowheads indicate nonglycosylated and glycosylated uncleaved isoform 2 *FAM19A5*, respectively.

The *FAM19A5* proteins corresponding to the 2 *FAM19A5* isoform transcripts differ in their N-terminal sequences (Fig. 3A). Isoform 1 produces a precursor protein that is cleaved at the end of the signal peptide to generate a mature, secreted 89-amino acid protein. Isoform 2, however, can have 2 different forms of proteins. Signal P analysis of the full amino acid sequence revealed a relatively low probability of a signal peptide at the N-terminus (Teufel et al., 2022). The presence of a stretch of hydrophobic α-helix in the N-terminus suggests that isoform 2 may produce an uncleaved membrane-associated protein consisting of 125 amino acids in size. Alternatively, the N-terminal sequence could function as a signal peptide, resulting in a secreted protein. Edman sequencing of the N-terminal residue of the secreted isoform 2 protein revealed the presence of pyroglutamate, followed by the Phe-Leu-Lys-Glu-Gly sequence. These findings suggest that the secreted isoform 2 protein is 100 amino acids in size (Fig. 3A). Therefore, this result differs from the findings of Wang et al. (2018), where the secreted isoform 2 protein claimed to be identical to the mature secreted protein of isoform 1.

We investigated the *FAM19A5* protein expression patterns in HEK293 cells expressing each type of *FAM19A5* isoform transcript. The isoform 1 protein was detected in the culture media at an expected molecular weight of approximately 12 kDa (Fig. 3B). In addition, higher molecular weight bands were observed, likely representing glycosylated forms of the protein due to modification at Asn113 (Fig. 3A). Isoform 1 proteins were also detected in the cell lysates with molecular weights similar to those observed in the media. Therefore, most of the intracellular isoform 1 protein is present in the cleaved form and is ready for secretion. Isoform 2 proteins were present in the media at a similar molecular weight to isoform 1. However, the isoform 2 protein is secreted less in quantity than the isoform 1 protein (Fig. 3B). However, in the cell lysate, isoform 2 proteins exhibited different molecular weights compared to those in the media. The lysate showed thick bands with higher molecular weights, along with a faint signal similar to that observed in the media. These observations indicate that the isoform 2 transcript produces 2 protein variants: a minor form that is cleaved at the signal P site and secreted outside the cell, and a major form that remains uncleaved, resulting in a higher molecular weight and retention within the cell (Fig. 3B). To determine whether the higher molecular weight bands represent glycosylated forms, we treated the *FAM19A5* proteins with PNGase F, an enzyme that deglycosylates proteins. The recombinant isoform 1 protein and the cell lysate isoform 2 proteins were treated with PNGase F, resulting in the disappearance of the higher molecular weight bands and an increase in the intensity of the lower molecular weight bands (Fig. 3C). This result suggests that both the secreted and uncleaved forms of the *FAM19A5* protein exist in either glycosylated or nonglycosylated states. Notably, the nonglycosylated secreted form of isoform 2 is slightly greater in molecular weight than isoform 1, as it contains an additional 11 amino acids (Fig. 3A).

### *FAM19A5* Protein Levels in the Brain and Peripheral Tissues

**Fig. 4:**
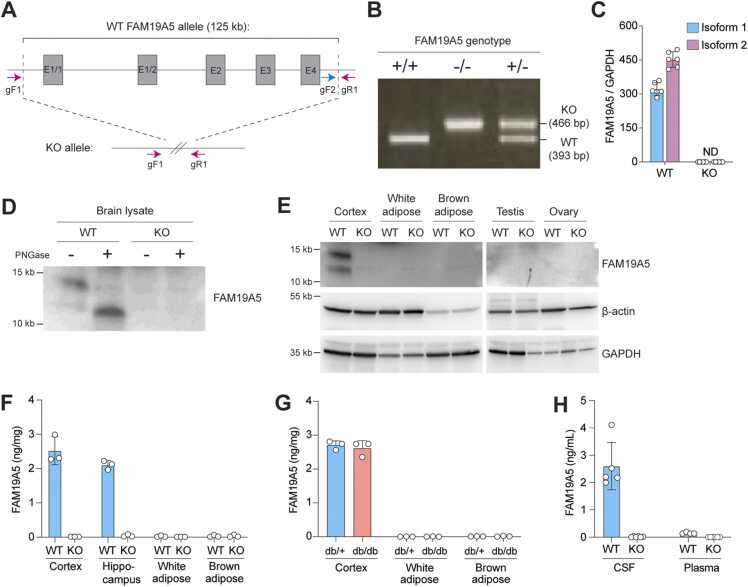
*FAM19A5* protein levels in WT, *FAM19A5* KO, and db/db mice. (A) A schematic diagram illustrating the genome structures of the WT and *FAM19A5* KO alleles. The *FAM19A5* gene from exon 1-1 (E1/1) to exon 4 (E4) was removed via the CRISPR/Cas9 system. The PCR primer locations used to determine the WT (gF1-gR1) and KO (gF2-gR1) alleles are indicated. (B) Genotyping of WT (+/+), heterozygous (+/−), and homozygous (−/−) *FAM19A5* KO mice using genomic DNA. (C) qRT-PCR analysis of the *FAM19A5* transcripts iso1 and iso2 in the cortex of WT and *FAM19A5* KO mice. (D) Western blot analysis of *FAM19A5* proteins extracted from the brains of WT and *FAM19A5* KO mice. The protein samples were treated with PNGase F to determine the presence of glycosylated forms of the *FAM19A5* protein. (E) Western blot analysis of *FAM19A5* proteins extracted from the cortex, white and brown adipose, testis, and ovary tissues of WT and *FAM19A5* KO mice. (F) Quantification of *FAM19A5* protein levels in the cortex, hippocampus, white adipose tissue, and brown adipose tissue of WT and *FAM19A5* KO mice via ELISA. (G) Quantification of *FAM19A5* protein levels in the cortex, white adipose tissue, and brown adipose tissue of db/db mice via ELISA. (H) Quantification of *FAM19A5* levels in the CSF and plasma of WT and *FAM19A5* KO mice.

*FAM19A5* KO mice were generated with the CRISPR/Cas9 system (Fig. 4A and B), and their *FAM19A5* expression levels were measured. qRT-PCR analysis revealed that the expression of the 1/2 isoform of *FAM19A5* was not detectable in brain tissue from the *FAM19A5* KO mice (Fig. 4C). We then investigated the presence of the *FAM19A5* protein in mouse brain extracts. Western blotting showed the presence of both glycosylated and nonglycosylated forms of *FAM19A5*. After treating the brain extracts with PNGase F, the intensity of the band corresponding to glycosylated *FAM19A5* greatly decreased, while the intensity of the band corresponding to nonglycosylated *FAM19A5* increased. Neither glycosylated nor nonglycosylated forms of the *FAM19A5* protein were observed in the brain extracts from *FAM19A5* KO mice (Fig. 4D).

Next, we measured the *FAM19A5* protein levels in white and brown adipose tissues. We also measured *FAM19A5* levels in the testis and ovary because they exhibited moderate levels of the *FAM19A5* transcript. None of the peripheral tissues presented detectable *FAM19A5* protein levels, which correlated with the measured *FAM19A5* transcript levels (Fig. 4E).

Notably, the *FAM19A5* proteins observed in the mouse tissue samples are likely the secreted form of isoform 1. The uncleaved isoform 2 protein, mainly observed in HEK293 cell lysates with a higher molecular weight than isoform 1, was not detected in either the brain or any peripheral tissues. Therefore, it is highly plausible that the nonsecreted form of isoform 2 observed in this study is an experimental artifact of the heterologous expression system. Alternatively, although the *FAM19A5* isoform 2 transcript is expressed in the brain and peripheral tissues, protein production from this isoform is likely negligible under physiological conditions.

We then investigated *FAM19A5* protein levels via ELISA, a more quantitative and sensitive method than Western blotting. In WT mice, approximately 2 to 3 ng/mg of *FAM19A5* was detected in the cortex and hippocampus of the brain. In contrast, *FAM19A5* was virtually undetectable in both tissues of *FAM19A5* KO mice. These findings are consistent with the Western blot results, confirming the reliability of ELISA in quantifying *FAM19A5*. In adipose tissues, only trace amounts of *FAM19A5* were detected, which were significantly lower than those in the brain. The levels in adipose tissues were similar to the background signal observed in KO mice, indicating negligible *FAM19A5* production in adipose tissues (Fig. 4F).

Previous studies have reported that *FAM19A5* protein levels decrease in metabolic dysfunctions such as obesity (Wang et al., 2018). To investigate whether this regulatory effect occurs, we quantified *FAM19A5* protein levels in white and brown adipose tissues of db/db mice, a model of obesity, via ELISA. In the cortex and hippocampus of db/+ and db/db mice, the *FAM19A5* protein levels were comparable to those of WT mice. Similar to WT mice, db/+ and db/db mice exhibited nearly negligible levels of the *FAM19A5* protein in the adipose tissues (Fig. 4G). As the *FAM19A5* levels in the adipose tissues were extremely low, the changes in the *FAM19A5* levels could not be accurately quantified.

We investigated the plasma and cerebrospinal fluid (CSF) levels of *FAM19A5* in WT and *FAM19A5* KO mice via ELISA. In WT mice, the levels of *FAM19A5* in CSF were detected at 2 to 4 ng/ml, whereas the levels in plasma were approximately 100 to 200 pg/ml, which is near the lowest level of the ELISA standard curve. In contrast, *FAM19A5* was not detected in the CSF or plasma of *FAM19A5* KO mice (Fig. 4H). This result further confirms the very low levels of plasma *FAM19A5* secreted from peripheral tissues, including adipose tissues.

### *FAM19A5* Does Not Interact With S1PR2

**Fig. 5:**
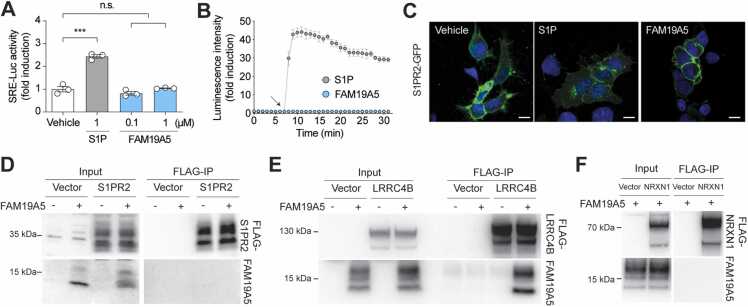
The *FAM19A5* protein did not interact with S1PR2. (A) An *SRE-luc* reporter assay was used to determine S1PR2 receptor activation in response to S1P and *FAM19A5*. The *SRE-luc* reporter and *S1PR2* genes were cotransfected into HEK293 cells that stably expressed the Gqi protein. The cells in serum-free medium were then treated with both S1P and *FAM19A5* for 6 h and subjected to a luciferase assay. (B) Nanoluc reporter assay for β-arrestin recruitment to S1PR2. The S1PR2-LgBit and β-arrestin-SmBit constructs were cotransfected into HEK293 cells. In a live-cell system, luminescence was determined before and after treatment with 1 µM S1P and *FAM19A5*. The data are presented as the means ± standard errors of the means from 3 independent experiments. (C) Internalization of S1PR2 in response to S1P and *FAM19A5*. HEK293 cells were transfected with the S1PR2-GFP construct. The cells were cultured under serum-free conditions for 16 h and then treated with 1 µM S1P and *FAM19A5*, for 30 min. The cellular locations of S1PR2-GFP in the presence of the ligands were determined via confocal microscopy. The scale bar represents 10 µm. (D-F) Co-IP of S1PR2 (D), LRRC4B (E), and NRXN1 (F) with recombinant *FAM19A5*. HEK293 cells were transfected with C-terminal FLAG-tagged S1PR2, LRRC4B, and NRXN1. The cells were then treated with 1 µM concentration of *FAM19A5* for 30 min. Following treatment, the cell lysates were immunoprecipitated using an anti-FLAG affinity gel, and subsequent immunoblot analysis was performed with both anti-FLAG and anti-*FAM19A5* antibodies.

A previous study reported that *FAM19A5* can regulate blood vessel smooth muscle function by interacting with S1PR2 (Wang et al., 2018). To investigate whether *FAM19A5* elicits S1PR2-mediated G-protein signaling, we used an SRE-luc assay system coupled to a HEK293Gqi stable cell line (Kim et al., 2014). Treatment with S1P, a natural agonist of S1PR2, for 6 h substantially increased SRE-luc activity, while treatment with *FAM19A5* did not (Fig. 5A). These data indicate that the *FAM19A5* protein cannot induce G-protein-mediated signal transduction in S1PR2-expressing cells.

Since some biased agonists induce β-arrestin recruitment to S1PR2 independently of G-protein-mediated signaling (Kim et al., 2014), we explored whether *FAM19A5* increased the interaction between S1PR2 and β-arrestin using the NanoLuc luciferase assay (Reyes-Alcaraz et al., 2018, Reyes-Alcaraz et al., 2019). S1P induced an immediate increase in luciferase activity, indicating an interaction between S1PR2 and β-arrestin. Conversely, *FAM19A5* had no effect (Fig. 5B).

Next, we investigated the effect of *FAM19A5* on the internalization of S1PR2. S1PR2 is localized primarily to the plasma membrane in the absence of ligand treatment. After treatment with S1P, the cellular internalization of S1PR2-GFP increased. However, treatment with *FAM19A5* did not result in S1PR2-GFP internalization (Fig. 5C).

We further investigated the physical interaction between S1PR2 and *FAM19A5* through a Co-IP experiment. Purified *FAM19A5* was added to HEK293 cells expressing FLAG-tagged S1PR2, followed by Co-IP using FLAG beads. The results showed that S1PR2 did not bind to *FAM19A5* (Fig. 5D). To validate the functional activity of the purified *FAM19A5* protein, we conducted a Co-IP experiment using HEK293 cells expressing FLAG-tagged LRRC4B. Recent studies have shown that *FAM19A5* interacts with LRRC4B, a postsynaptic adhesion protein, likely contributing to synapse disassembly (Kim et al., 2024). The Co-IP results demonstrated that FLAG-tagged LRRC4B is effectively bound to *FAM19A5* (Fig. 5E). We then investigated whether *FAM19A5* binds to NRXN1, a presynaptic adhesion molecule known to interact with FAM19A1, A2, A3, and A4, but not with *FAM19A5* (Khalaj et al., 2020). The Co-IP experiments revealed that FLAG-tagged NRXN1 did not bind to *FAM19A5* (Fig. 5F). These findings demonstrate that *FAM19A5* specifically binds to LRRC4B, but not to S1PR2 or NRXN1. Collectively, these data indicate that *FAM19A5* cannot induce either β-arrestin recruitment to S1PR2 or ligand-stimulated internalization of S1PR2.

## DISCUSSION

The quantitative levels of total *FAM19A5* transcripts, isoform 1, and isoform 2 were investigated in a wide variety of peripheral tissues and compared with the levels in specific brain regions. In general, *FAM19A5* transcript levels in peripheral tissues are very low compared with those in brain tissues. Isoform 1 expression was minimal in almost all the peripheral tissues examined in this study. While the isoform 2 transcript was expressed to some extent in certain peripheral tissues, such as reproductive organs and adipose tissues, the protein levels in these peripheral tissues were nearly undetectable, as determined by Western blotting and ELISA.

Similarly, *FAM19A5* promoter-driven X-gal signals were also very faint or negligible in peripheral tissues compared with the signal in the brain. Only a few samples exhibited faint-to-moderate X-gal signals, including cardiac muscle cells, gastrointestinal cells in the muscularis externa, and germ cells in the testis and ovary. We did not detect X-gal signals in any other tissues, including white adipose tissue and the thyroid and adrenal glands.

Quantitative RT-PCR, X-gal staining in the *FAM19A5-LacZ* KI mice, Western blotting, and ELISA consistently demonstrated extremely low levels of *FAM19A5* expression in adipose tissues. This finding contrasts with a previous report showing that these tissues produce significantly high levels of both *FAM19A5* transcripts and protein (Wang et al., 2018). In the referenced study, the authors investigated *FAM19A5* levels in human adipose tissues using qRT-PCR and immunohistochemistry, as well as determined the levels of *FAM19A5* secreted by various cell types via ELISA (Wang et al., 2018). Therefore, differences in species (human vs mouse) and methodologies may account for the discrepancies in the results. However, recent public databases, such as the Human Protein Atlas, indicate that *FAM19A5* transcript levels in human adipose tissues are approximately 1/100 to 5/100 of those found in the human brain (Karlsson et al., 2021, Sjöstedt et al., 2020). These datasets also show extremely low levels of *FAM19A5* transcripts in most peripheral tissues and low-to-moderate expression levels in both female and male reproductive tissues. These findings are more consistent with our qRT-PCR and *LacZ* expression data than those reported by Wang et al. (2018). Regarding the methods used to analyze *FAM19A5* protein expression, our Western blot and ELISA were validated by comparing *FAM19A5* expression in WT and *FAM19A5* KO mice. Both Western blot and ELISA revealed the absence of the *FAM19A5* protein in the KO mice. Western blot showed that the *FAM19A5* protein levels in the adipose and reproductive tissues of WT mice were below detectable limits, whereas its expression in brain tissue was high. ELISA also revealed negligible levels of *FAM19A5* in the adipose tissues of both WT and db/db mice.

Regarding *FAM19A5* levels in the blood, Wang et al. (2018) reported that the concentration of *FAM19A5* in the blood of normal mice was approximately 400 ng/ml. This concentration is significantly higher than those of other cytokines, chemokines, and hormones, which typically range between 10 and 20 ng/ml (Burnett et al., 2017, Hillyer and Woodward, 2003, Kapilevich et al., 2017, Nilsson et al., 2015, Paudel et al., 2019, Zhao et al., 2019). Our ELISA analysis revealed that the plasma concentration of the *FAM19A5* protein in WT mice was less than 200 pg/ml, while its concentration in CSF was significantly higher, ranging from 2 to 4 ng/ml. The much higher concentration of *FAM19A5* in the CSF compared with blood suggests that *FAM19A5* functions primarily within the brain rather than in peripheral tissues.

Recent findings suggest that *FAM19A5* interacts with LRRC4B, a postsynaptic adhesion molecule, while its paralogs FAM19A1, A2, A3, and A4 interact with NRXN1, a presynaptic adhesion molecule (Khalaj et al., 2020, Kim et al., 2024). Therefore, *FAM19A5* and its paralogous proteins are likely involved in synaptic homeostasis, although their binding partners differ. Co-IP experiments demonstrated that purified *FAM19A5* binds to LRRC4B but not to NRXN1 or S1PR2. Additionally, the *FAM19A5* protein did not increase S1PR2-mediated signaling events, such as G-protein-mediated signaling, β-arrestin recruitment, or receptor internalization, whereas its authentic ligand S1P activated S1PR2.

In summary, this study thoroughly analyzed *FAM19A5* expression across various peripheral tissues and compared it with its expression in the brain by examining *FAM19A5* transcript levels, *LacZ* expression in *FAM19A5-LacZ* KI mice, tissue-specific *FAM19A5* protein content, and concentrations of secreted *FAM19A5* in biofluids such as blood and CSF. The results consistently demonstrated significantly higher *FAM19A5* expression in the brain compared with extremely low levels in peripheral tissues, including adipose tissues. Given the low levels of the *FAM19A5* protein in peripheral tissues and blood, further research is needed to understand its functional role in these tissues under both normal and pathological conditions.

## Author Contributions

J.Y.S., H.Y.K., E.H.C., W.S.L., and S.J.Y. wrote the paper. H.Y.K., E.H.C., E.B.C., Y.N.L., M.H.L., N.H., and S.T.O. performed the experiments. J.Y.S., H.Y.K., and E.H.C. analyzed the data. A.S., H.J.Y., A.R.A., Y.W.J., Y.R.L., W.K.K., and S.G.K. provided reagents and samples. S.Y.S., J.K.L., and J.I.H provided expertise and feedback. J.Y.S. supervised the research and secured funding.

## Materials and Correspondence

Correspondence and material requests should be addressed to J.Y.S. (jyseong@korea.ac.kr).

## Declaration of Competing Interests

The authors declare the following financial interests/personal relationships that may be considered as potential competing interests: Hoyun Kwak, Eun-Ho Cho, Eun Bee Cho, Yongwoo Jeong, Yerim Lee, Minhyeok Lee, Nui Ha, Sitaek Oh, Jae Keun Lee, Won Suk Lee, Won Kyum Kim, Sangjin Yoo, Soon-Gu Kwon, and Jae Young Seong are employed by Neuracle Science Co, Ltd. Anu Shahapal, Hyo Jeong Yong, Arfaxad Reyes-Alcaraz, Yongwoo Jeong, Minhyeok Lee, Won Kyum Kim, Soon-Gu Kwon, and Jae Young Seong are shareholders of Neuracle Science Co, Ltd. Yoo-Na Lee and Jong-Ik Hwang have no conflicts of interest to declare.

## Data Availability

The raw data and genetic constructs are available upon request to the authors.

## ORCID

Eun-Ho Cho: https://orcid.org/0000-0001-6043-6189

Hoyun Kwak: https://orcid.org/0000-0002-9679-521X

Eun Bee Cho: https://orcid.org/0009-0005-3326-9692

Yoo-Na Lee: https://orcid.org/0000-0002-9590-236X

Hyo Jeong Yong: https://orcid.org/0000-0002-9172-3233

Anu Shahapal: https://orcid.org/0000-0002-0602-2898

Arfaxad Reyes-Alcaraz: https://orcid.org/0000-0002-2650-8430

Yongwoo Jeong: https://orcid.org/0000-0003-1358-0123

Yerim Lee: https://orcid.org/0009-0003-4822-4142

Minhyeok Lee: https://orcid.org/0009-0003-0345-1456

Nui Ha: https://orcid.org/0009-0005-9865-0179

Sitaek Oh: https://orcid.org/0009-0000-0438-1267

Won Suk Lee: https://orcid.org/0000-0001-5543-3477

Won Kyum Kim: https://orcid.org/0009-0004-4011-3517

Sangjin Yoo: https://orcid.org/0000-0002-0449-4242

Soon-Gu Kwon: https://orcid.org/0009-0006-0000-2163

Jong-Ik Hwang: https://orcid.org/0000-0002-0729-1782

Jae Young Seong: https://orcid.org/0000-0002-3312-6924
