# Supplementary material for: Is FAM19A5 an adipokine? Peripheral FAM19A5 in wild-type, FAM19A5 knockout, and LacZ knockin mice
Source: Mol Cells. 2024 Oct 18;47(12):100125. doi: 10.1016/j.mocell.2024.100125 (PMC11603079; doi:10.1016/j.mocell.2024.100125)
Supplement: Supplementary file 1 — Supplementary material [file mmc1.docx]

**Supplementary Materials**

**Is FAM19A5 an adipokine? Peripheral FAM19A5 in wild-type, FAM19A5 knockout, and LacZ knockin mice**

Hoyun Kwak^1,4^, Eun-Ho Cho^1,4^, Eun Bee Cho^1^, Yoo-Na Lee^2^, Anu Shahapal^2^, Hyo Jeong Yong^2^, Arfaxad Reyes-Alcaraz^3^, Yongwoo Jeong^1^, Yerim Lee^1^, Minhyeok Lee^1^, Nui Ha^1^, Sitaek Oh^1^, Jae Keun Lee^1^, Won Suk Lee^1^, Won Kyum Kim^1^, Sangjin Yoo^1^, Soon-Gu Kwon^1^, Jong-Ik Hwang^2^ & Jae Young Seong^1,2,*^

^1^Neuracle Science Co. Ltd., Seoul 02841, Republic of Korea

^2^Graduate School of Biomedical Sciences, Korea University College of Medicine, Seoul 02841, Republic of Korea

^3^College of Pharmacy, University of Houston, Texas 77204, United States of America

*Corresponding author.

Tel: +82-2-2286-1090; Fax: +82-2-921-4355; E-mail: [jyseong@korea.ac.kr](mailto:jyseong@korea.ac.kr)

^4^ These authors contributed equally to this work.

**Supplementary Table 1. X-gal staining of peripheral tissues**

| Peripheral organ/tissue | | | Group | | | | | | | |
| --- | --- | --- | --- | --- | --- | --- | --- | --- | --- | --- |
|  |  |  | WT | | | *Hetero*  (+/-) | *Homozygote*  (+/+) | | | |
|  |  |  | Male#1 | Male#2 | Female#1 | Male#1 | Male#1 | Male#2 | Male#3 | Female #1 |
| Heart | Cardiac muscle cell | | - | - | N.T. | 1+ | 2+ | 1+ | 1+ | 2+ |
| Stomach | Mucosa | Gastric gland | Δ | Δ | N.T. | Δ | Δ | Δ | Δ | N.T. |
|  | Submucosa | Connective tissue | - | - | N.T. | - | - | - | - | N.T. |
|  | Muscularis  externa | Smooth muscle & Myenteric plexus | - | - | N.T. | 1+ | 1+ | - | 1+ | 1+ |
| Small intestine | Mucosa | Villus & Crypt | - | Δ | N.T. | - | - | - | - | - |
|  | Submucosa | Connective tissue | - | - | N.T. | - | - | - | - | - |
|  | Muscularis externa | Smooth muscle & Myenteric plexus | - | - | N.T. | 1+ | 1+ | 1+ | 1+ | 1+ |
| Large intestine | Mucosa | Villus & Crypt | Δ | Δ | N.T. | Δ | Δ | Δ | Δ | Δ |
|  | Submucosa | Connective tissue | - | - | N.T. | - | - | - | N.T. | - |
|  | Muscularis externa | Smooth muscle & Myenteric plexus | - | - | N.T. | 1+ | 1+ | 1+ | N.T. | 1+ |
| Testis | Seminiferous tubule | Spermatogenic cells | - | - | N.A. | 2+ | N.T. | 3+ | 3+ | N.A. |
|  | Interstitium | Leydig cell | Δ | Δ | N.A. | Δ | N.T. | Δ | Δ | N.A. |
| Ovary | Ovarian follicle | Oocyte | N.A. | N.A. | - | N.A. | N.A. | N.A. | N.A. | 1+ |
|  |  | Granulosa cell | N.A. | N.A. | Δ | N.A. | N.A. | N.A. | N.A. | Δ |
|  | Corpus luteum | Lutein cell | N.A. | N.A. | Δ | N.A. | N.A. | N.A. | N.A. | Δ |
| Uterus | Endometrium | Endometrial gland | N.A. | N.A. | Δ | N.A. | N.A. | N.A. | N.A. | Δ |
|  |  | Endometrial stroma | N.A. | N.A. | - | N.A. | N.A. | N.A. | N.A. | - |
|  | Myometrium | Smooth muscle cell | N.A. | N.A. | - | N.A. | N.A. | N.A. | N.A. | 1+ |
| Adrenal gland | Cortex | | - | N.T. | - | - | - | N.T. | N.T. | - |
|  | Medulla | | - | N.T. | - | - | - | N.T. | N.T. | - |
|  | X-Zone | | N.A. | N.A. | - | N.A. | N.A. | N.A. | N.A. | 1+ |
| Aorta | Endothelium | | N.T. | - | N.T. | N.T. | N.T. | - | - | N.T. |
|  | Smooth muscle & Connective tissue | | N.T. | - | N.T. | N.T. | N.T. | - | - | N.T. |
| Kidney | Glomerulus | | Δ | Δ | N.T. | Δ | Δ | Δ | Δ | Δ |
|  | Renal tubule | | Δ | Δ | N.T. | Δ | Δ/+ | Δ | Δ | Δ/+ |
| Lung | Bronchiole epithelium | | Δ | N.T. | N.T. | Δ | Δ | N.T. | N.T. | - |
|  | Pneumocyte | | - | N.T. | N.T. | - | - | N.T. | N.T. | - |
|  | Alveolar macrophage | | Δ | N.T. | N.T. | Δ | Δ | N.T. | N.T. | - |
| Pancreas | Exocrine gland | | - | N.T. | N.T. | - | - | N.T. | N.T. | - |
|  | Endocrine gland (pancreatic islet) | | - | N.T. | N.T. | - | - | N.T. | N.T. | - |
| Liver | Hepatocyte | | - | N.T. | N.T. | - | - | N.T. | N.T. | - |
|  | Bile duct | | - | N.T. | N.T. | - | - | N.T. | N.T. | - |
|  | Central vein | | - | N.T. | N.T. | - | - | N.T. | N.T. | - |
|  | Portal vein | | - | N.T. | N.T. | - | - | N.T. | N.T. | - |
| Submandibular  gland | Serous acinus | | N.T. | - | N.T. | N.T. | N.T. | - | - | N.T. |
|  | Mucous acinus | | N.T. | Δ | N.T. | N.T. | N.T. | Δ | Δ | N.T. |
|  | Connective tissue | | N.T. | - | N.T. | N.T. | N.T. | - | - | N.T. |
| Sublingual  gland | Mucous acinus | | N.T. | Δ | N.T. | N.T. | N.T. | N.T. | Δ | N.T. |
|  | Connective tissue | | N.T. | - | N.T. | N.T. | N.T. | N.T. | - | N.T. |
| Parotid gland | Serous acinus | | N.T. | Δ | N.T. | N.T. | N.T. | Δ | Δ | N.T. |
|  | Connective tissue | | N.T. | - | N.T. | N.T. | N.T. | - | - | N.T. |
| Thymus | Cortex | | Δ | N.T. | N.T. | Δ | Δ | N.T. | N.T. | Δ |
|  | Medulla | | Δ | N.T. | N.T. | Δ | Δ | N.T. | N.T. | Δ |
| Spleen | White pulp | | Δ | N.T. | N.T. | Δ | Δ | N.T. | N.T. | Δ |
|  | Red pulp | | Δ | N.T. | N.T. | Δ | Δ | N.T. | N.T. | Δ |
| Bone marrow | Hematopoietic cell | | - | N.T. | N.T. | - | - | N.T. | N.T. | - |
| Pituitary gland | Pars nervosa | | N.T. | - | N.T. | N.T. | N.T. | - | - | N.T. |
|  | Pars intermediate | | N.T. | - | N.T. | N.T. | N.T. | - | - | N.T. |
|  | Pars distalis | | N.T. | - | N.T. | N.T. | N.T. | - | N.T. | N.T. |
| Thyroid gland | Follicular cell | | N.T. | Δ | N.T. | N.T. | N.T. | N.T. | Δ | N.T. |
|  | Connective tissue | | N.T. | - | N.T. | N.T. | N.T. | N.T. | - | N.T. |
| Skeletal muscle | | | - | N.T. | N.T. | - | - | N.T. | N.T. | - |
| White adipose  tissue | White adipocyte | | - | N.T. | N.T. | N.T. | - | N.T. | N.T. | - |
|  | Connective tissue | | - | N.T. | N.T. | N.T. | - | N.T. | N.T. | - |
| Brown adipose  tissue | Brown adipocyte | | - | N.T. | N.T. | - | - | N.T. | N.T. | - |
|  | Connective tissue | | - | N.T. | N.T. | - | - | N.T. | N.T. | - |
| Skin | Epidermis | Stratified squamous epithelium | N.T. | - | N.T. | N.T. | N.T. | - | - | N.T. |
|  | Dermis | Hair follicle | N.T. | - | N.T. | N.T. | N.T. | - | - | N.T. |
|  |  | Sebaceous gland | N.T. | Δ | N.T. | N.T. | N.T. | Δ | Δ | N.T. |
|  |  | Connective tissue | N.T. | - | N.T. | N.T. | N.T. | - | - | N.T. |
|  | Hypodermis | White adipose tissue | N.T. | - | N.T. | N.T. | N.T. | - | - | N.T. |
|  |  | Striated muscle cell | N.T. | - | N.T. | N.T. | N.T. | - | - | N.T. |
|  |  | Peripheral nerve | N.T. | - | N.T. | N.T. | N.T. | - | - | N.T. |
| Seminal vesicle | Pseudostratified columnar epithelium | | - | - | N.T. | N.T. | - | - | - | N.T. |
| Coagulating  gland | Simple cuboidal to columnar epithelium | | Δ | Δ | N.T. | N.T. | N.T. | Δ | Δ | N.T. |

Ten-week-old adult wild-type littermates (2 males and 1 female) and *FAM19A5-LacZ* KI heterozygote (1 male) and homozygote (3 males and 1 female) mice were subjected to X-gal staining. The criteria for grading the X-gal signal are described in the Materials and Methods section. N.T.: Not Tested. N.A.: Not Applicable.

**Supplementary Table 2. Comparison of FAM19A5 RNA expression profiles across mouse and human databases**

| Peripheral  tissues | Mouse | | | | Human | | | | | |
| --- | --- | --- | --- | --- | --- | --- | --- | --- | --- | --- |
|  | qRT-PCR | | BioGPS | | HPA | | GTEx | | FANTOM5 | |
|  | FAM19A5  /GAPDH | Rel. Value | nTPM | Rel. Value | nTPM | Rel. Value | nTPM | Rel. Value | TPM | Rel. Value |
| Cortex | 929.7 | 100 | 413.5 | 100 | 24.6 | 100 | 78.2 | 100 | 44.8 | 100 |
| Hippocampus | 1266.9 | 136.3 | 819.8 | 198.26 | N.T. | - | 82.8 | 105.9 | 27.9 | 62.3 |
| Spinal cord | 887.8 | 95.5 | 821.8 | 198.75 | N.T. | - | 36.2 | 46.3 | 15.8 | 35.3 |
| DRG | 230.3 | 24.8 | 146.7 | 35.48 | N.T. | - | N.T. | - | N.T. | - |
| Heart | 20.6 | 2.2 | 22.8 | 5.51 | 0.5 | 2.0 | 2.5 | 3.2 | 0.3 | 0.7 |
| Aorta | 16.0 | 1.7 | N.T. | - | N.T. | - | N.T. | - | N.T. | - |
| Kidney | 36.7 | 3.9 | 7.7 | 1.86 | 0.9 | 3.7 | 2.3 | 2.9 | N.D. | - |
| Lung | 29.8 | 3.2 | 4.8 | 1.15 | 1 | 4.1 | 2 | 2.6 | 1.1 | 2.5 |
| Stomach | 31.8 | 3.4 | 7.1 | 1.72 | 1.9 | 7.7 | 2.8 | 3.6 | N.T. | - |
| Small Intestine | 3.9 | 0.4 | 5.1 | 1.23 | 1.2 | 4.9 | 3.2 | 4.1 | 0.3 | 0.7 |
| Large Intestine | 31.2 | 3.4 | 8.1 | 1.95 | 0.5 | 2.0 | 8.1 | 10.4 | 0.7 | 1.6 |
| Pancreas | 34.0 | 3.7 | 8.5 | 2.05 | 1.2 | 4.9 | 2.3 | 2.9 | N.D. | - |
| Liver | 6.9 | 0.8 | 6.0 | 1.46 | 1.7 | 6.9 | 2.7 | 3.5 | 1.5 | 3.3 |
| Salivary gland | 19.7 | 2.1 | 21.6 | 5.23 | 4.7 | 19.1 | 4.6 | 5.9 | 7.4 | 16.5 |
| Thymus | 7.6 | 0.8 | N.T. | - | 1.1 | 4.5 | N.T. | - | 3.5 | 7.8 |
| Spleen | 9.9 | 1.1 | 4.6 | 1.12 | 0.3 | 1.2 | 0.3 | 0.4 | N.D. | - |
| Bone marrow | 12.7 | 1.4 | 4.7 | 1.14 | N.D. | - | N.T. | - | N.T. | - |
| Adrenal gland | 81.1 | 8.7 | 45.4 | 10.99 | 1.2 | 4.9 | 1.1 | 1.4 | N.T. | - |
| Pituitary gland | 138.6 | 14.9 | 57.9 | 14.01 | N.T. | - | 6.8 | 8.7 | 1.4 | 3.1 |
| Thyroid gland | 43.0 | 4.6 | N.T. | - | 0.2 | 0.8 | 0.8 | 1.0 | 0.5 | 1.1 |
| Skeletal muscle | 4.2 | 0.5 | 16.9 | 4.10 | 0.6 | 2.4 | 1.4 | 1.8 | 1.1 | 2.5 |
| White adipose | 38.8 | 4.2 | 5.00 | 1.21 | 0.8 | 3.3 | 2.6 | 3.3 | 0.6 | 1.3 |
| Brown adipose | 7.9 | 0.9 | 11.8 | 2.84 | N.T. | - | N.T. | - | N.T. | - |
| Skin | 28.9 | 3.1 | N.T. | - | N.T. | - | N.T. | - | N.T. | - |
| Testis | 45.1 | 4.9 | 4.7 | 1.14 | 1.2 | 4.9 | 3.3 | 4.2 | 2 | 4.5 |
| Seminal vesicle | 115.3 | 12.4 | N.T. | - | 3.1 | 12.6 | N.T. | - | 1.2 | 2.7 |
| Coagulating gland | 154.9 | 16.7 | N.T. | - | N.T. | - | N.T. | - | N.T. | - |
| Ovary | 137.3 | 14.8 | 17.1 | 4.14 | 7.7 | 31.3 | 11.7 | 15.0 | 4.6 | 10.3 |
| Uterus | 172.9 | 18.6 | 11.0 | 2.65 | N.T. | - | N.T. | - | N.T. | - |

The qRT-PCR results represent the mean value of the total FAM19A5 transcript level, as shown in Table 1. The FAM19A5 RNA expression level is presented as TPM (transcripts per million) values, derived from publicly available tissue RNA-seq data from BioGPS, HPA (Human Protein Atlas), GTEx, and FANTOM5. The relative (Rel) values for each analysis were normalized to the expression level in the cortex. N.T.: Not Tested., N.D.: Not Detected.

**Supplementary Fig. 1. X-gal signal in heart.**


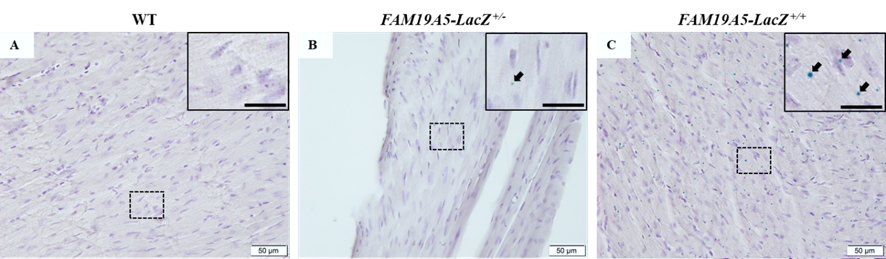


Representative light photomicrographs of heart cryosections of (A) wild type (WT, male #1), (B) *FAM19A5-LacZ*^+/-^ (heterozygote, male #1), and (C) *FAM19A5-LacZ*^+/+^ (homozygote, male #1) mice. The cryosections were stained with X-gal solution and counterstained with hematoxylin. The image in the dashed box is magnified in the inset, which is outlined in the same color as the dashed box. The black arrows indicate punctate blue precipitates in cardiac muscle cells observed only in *FAM19A5-LacZ* KI mice but not in WT mice. The scale bars in the inset represent 20 μm.

**Supplementary Fig. 2. X-gal signal in stomach.**


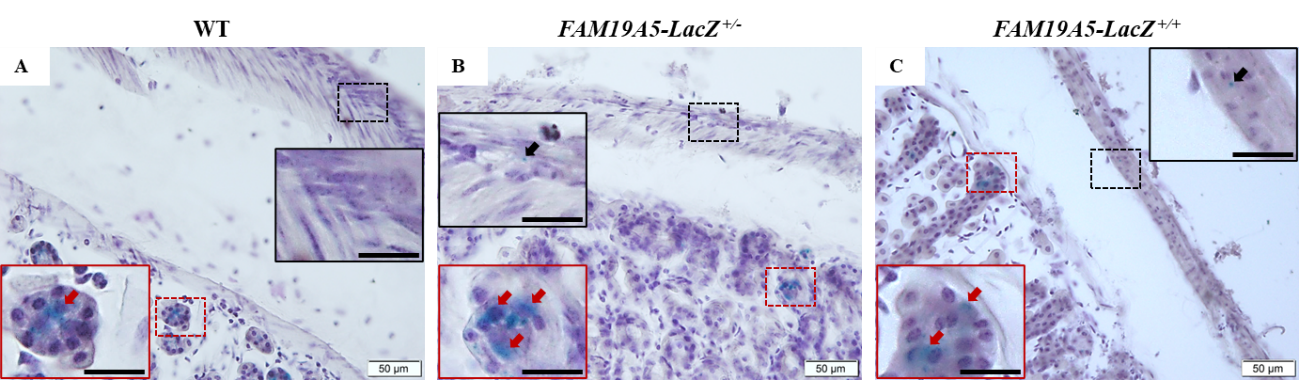


Representative light photomicrographs of stomach cryosections of (A) wild type (WT, male #1), (B) *FAM19A5-LacZ*^+/-^ (heterozygote, male #1), and (C) *FAM19A5-LacZ*^+/+^ (homozygote, male #3) mice. The cryosections were stained with X-gal solution and counterstained with hematoxylin. The image in the dashed box is magnified in the inset, which is outlined in the same color as the dashed box. The black arrows indicate punctate blue precipitates in smooth muscle cells or myenteric plexuses observed only in *FAM19A5-LacZ* KI mice but not in WT mice. Red arrows indicate dispersed blue precipitates in gastric glands observed in both WT and *FAM19A5-LacZ* KI mice. The scale bars in the inset represent 20 μm.

**Supplementary Fig. 3. X-gal signal in small intestine.**


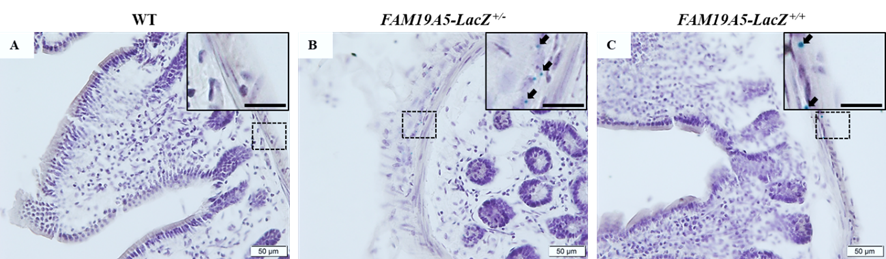


Representative light photomicrographs of small intestine cryosections of (A) wild type (WT, male #1), (B) *FAM19A5-LacZ*^+/-^ (heterozygote, male #1), and (C) *FAM19A5-LacZ*^+/+^ (homozygote, male #1) mice. The cryosections were stained with X-gal solution and counterstained with hematoxylin. The image in the dashed box is magnified in the inset, which is outlined in the same color as the dashed box. The black arrows indicate punctate blue precipitates in smooth muscle cells or myenteric plexuses observed only in *FAM19A5-LacZ* KI mice but not in WT mice. The scale bars in the inset represent 20 μm.

**Supplementary Fig. 4. X-gal signal in large intestine.**


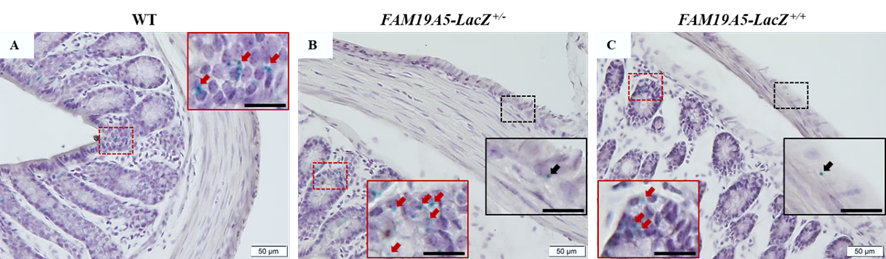


Representative light photomicrographs of large intestine cryosections of (A) wild type (WT, male #1), (B) *FAM19A5-LacZ*^+/-^ (heterozygote, male #1), and (C) *FAM19A5-LacZ*^+/+^ (homozygote, male #1) mice. The cryosections were stained with X-gal solution and counterstained with hematoxylin. The image in the dashed box is magnified in the inset, which is outlined in the same color as the dashed box. The black arrows indicate punctate blue precipitates in smooth muscle cells or myenteric plexuses observed only in *FAM19A5-LacZ* KI mice but not in WT mice. Red arrows indicate punctate blue precipitates in mucosa observed in both WT and *FAM19A5-LacZ* KI mice. The scale bars in the inset represent 20 μm.

**Supplementary Fig. 5. X-gal signal in testis.**


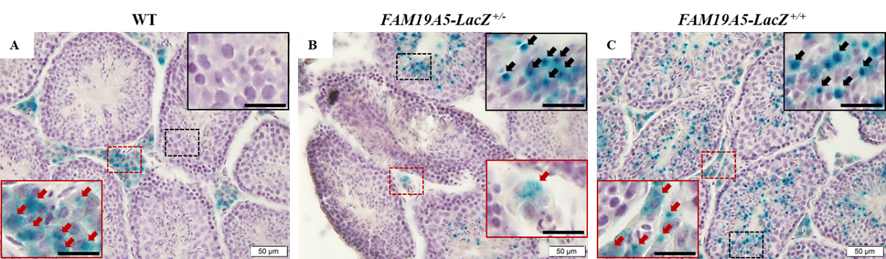


Representative light photomicrographs of testis cryosections of (A) wild type (WT, male #2), (B) *FAM19A5-LacZ*^+/-^ (heterozygote, male #1), and (C) *FAM19A5-LacZ*^+/+^ (homozygote, male #2) mice. The cryosections were stained with X-gal solution and counterstained with hematoxylin. The image in the dashed box is magnified in the inset, which is outlined in the same color as the dashed box. The black arrows indicate punctate blue precipitates in germ cells observed only in *FAM19A5-LacZ* KI mice but not in WT mice. Red arrows indicate dispersed blue precipitates in interstitial cells (Leydig cells) observed in both WT and *FAM19A5-LacZ* KI mice. The scale bars in the inset represent 20 μm.

**Supplementary Fig. 6. X-gal signal in ovary.**


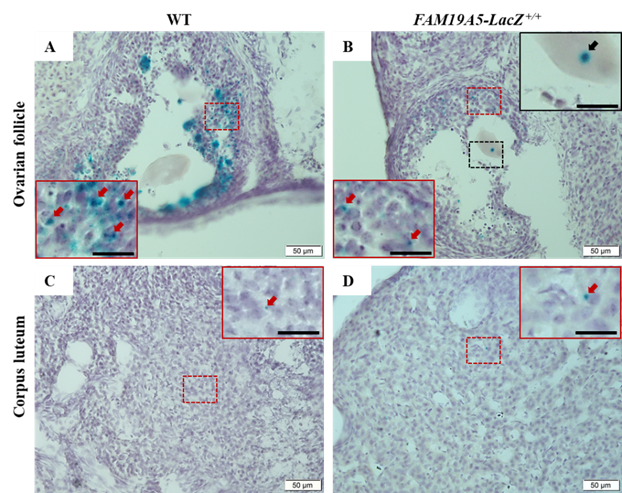


Representative light photomicrographs of ovary cryosections of (A, C) wild-type (WT, female #1) and (B, D) *FAM19A5-LacZ*^+/+^ (homozygote, female #1) mice. Ovarian follicles (A-B) and corpus luteum (C-D) were analyzed. The cryosections were stained with X-gal solution and counterstained with hematoxylin. The image in the dashed box is magnified in the inset, which is outlined in the same color as the dashed box. The black arrows indicate punctate blue precipitates in oocytes of *FAM19A5-LacZ* KI mice but not in WT mice. Red arrows indicate punctate/dispersed blue precipitates in granulosa and lutein cells observed in both WT and *FAM19A5-LacZ*^+/+^ mice. The scale bars in the inset represent 20 μm.

**Supplementary Fig. 7. X-gal signal in uterus.**


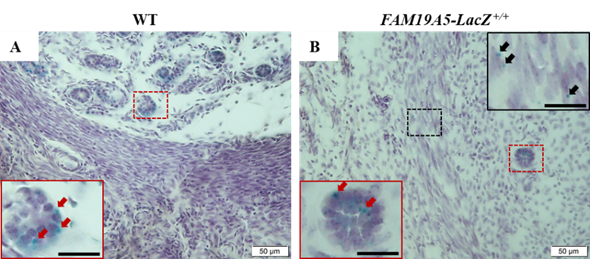


Representative light photomicrographs of uterus cryosections of (A) wild type (WT, female #1) and (B) *FAM19A5-LacZ*^+/+^ (homozygote, female #1) mice. The cryosections were stained with X-gal solution and counterstained with hematoxylin. The image in the dashed box is magnified in the inset, which is outlined in the same color as the dashed box. The black arrows indicate punctate blue precipitates in myometrial smooth muscle cells observed only in *FAM19A5-LacZ*^+/+^ mouse but not in WT mouse. Red arrows indicate punctate/dispersed blue precipitates in the endometrial gland observed in both WT and *FAM19A5-LacZ*^+/+^ mice. The scale bars in the inset represent 20 μm.

**Supplementary Fig. 8. X-gal signal in adrenal gland.**


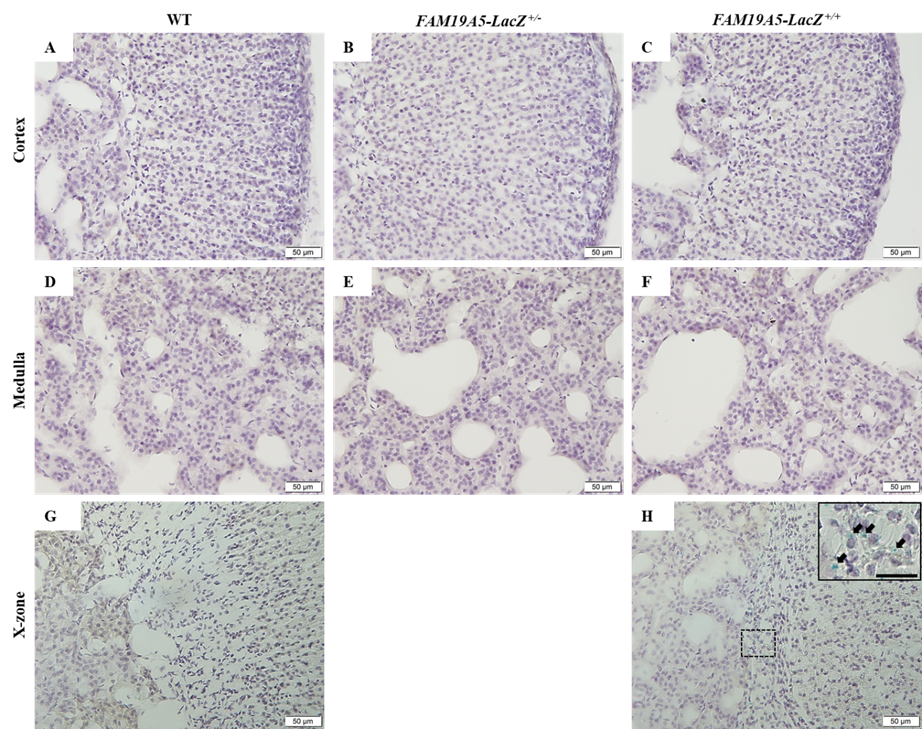


Representative light photomicrographs of adrenal gland cryosections of (A, D, G) wild type (WT, male #1 and female #1), (B, E) *FAM19A5-LacZ*^+/-^ (heterozygote, male #1), and (C, F, H) *FAM19A5-LacZ*^+/+^ (homozygote, male #1 and female #1) mice. The adrenal cortex (A-C), medulla (D-F) and X-zone, located at the junction of the cortex and the medulla (G-H), were examined. Cryosections were stained with X-gal solution and counterstained with hematoxylin. No X-gal signals were detected in the adrenal cortex or medulla of WT or *FAM19A5-LacZ* mice. The image in the dashed box is magnified in the inset, which is outlined in the same color as the dashed box. The black arrows indicate punctate blue precipitates in the X-Zone were observed in *FAM19A5-LacZ*^+/+^ mice but not in WT mice. The scale bars in the inset represent 20 μm.

**Supplementary Fig. 9. X-gal signal in aorta.**


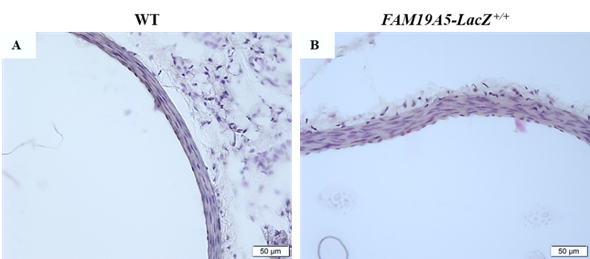


Representative light photomicrographs of thoracic (descending) aorta cryosections of (A) wild type (WT, male #2) and (B) *FAM19A5-LacZ*^+/+^ (homozygote, male #2) mice. The cryosections were stained with X-gal solution and counterstained with hematoxylin. No X-gal signals were detected in either WT or *FAM19A5-LacZ*^+/+^ mice.

**Supplementary Fig. 10. X-gal signal in kidney.**


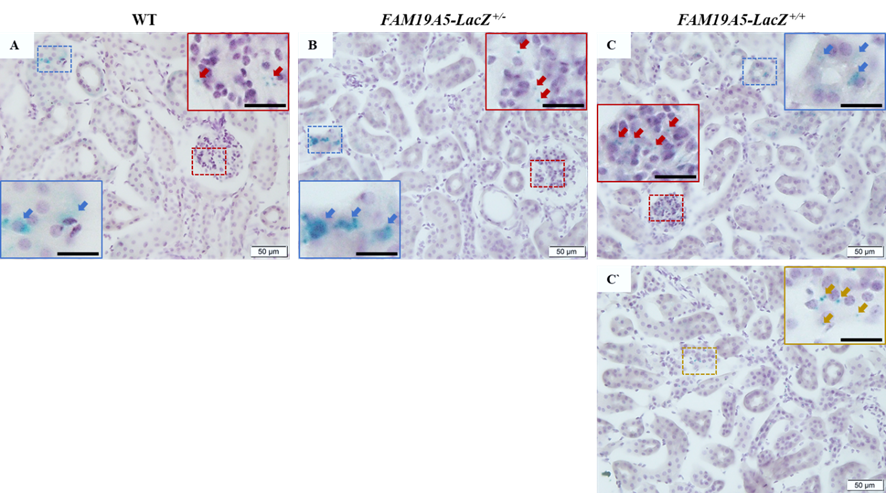


Representative light photomicrographs of kidney cryosections of (A) wild type (WT, male #2), (B) *FAM19A5-LacZ*^+/-^ (heterozygote, male #1), and (C and C`) *FAM19A5-LacZ*^+/+^ (homozygote, male #1) mice. The cryosections were stained with X-gal solution and counterstained with hematoxylin. The image in the dashed box is magnified in the inset, which is outlined in the same color as the dashed box. Red arrows indicate punctate blue precipitates in glomeruli observed in both WT and *FAM19A5-LacZ* KI mice. The blue arrows indicate dispersed blue precipitates in renal tubular epithelia observed in both WT and *FAM19A5-LacZ* KI mice. Yellow arrows indicate punctate blue precipitates in renal tubular epithelia observed only in *FAM19A5-LacZ* KI mice but not in WT mice. The scale bars in the inset represent 20 μm.

**Supplementary Fig. 11. X-gal signal in lung.**


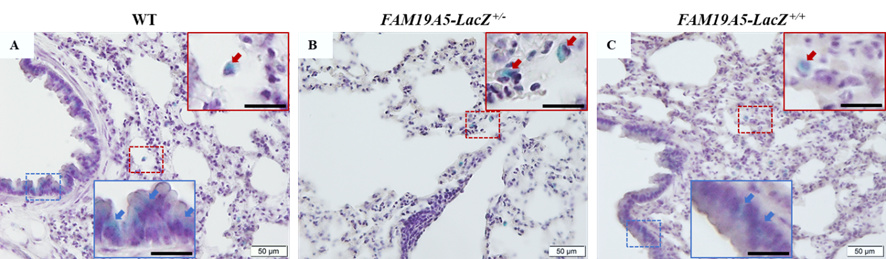


Representative light photomicrographs of lung cryosections of (A) wild type (WT, male #1), (B) *FAM19A5-LacZ*^+/-^ (heterozygote, male #1), and (C) *FAM19A5-LacZ*^+/+^ (homozygote, male #1) mice. The cryosections were stained with X-gal solution and counterstained with hematoxylin. The image in the dashed box is magnified in the inset, which is outlined in the same color as the dashed box. Red arrows indicate dispersed blue precipitates in alveolar macrophages observed in both WT and *FAM19A5-LacZ* mice. The blue arrows indicate dispersed blue precipitates in bronchiole epithelia (Simple columnar or cuboidal epithelia) observed in both WT and *FAM19A5-LacZ* KI mice. The scale bars in the inset represent 20 μm.

**Supplementary Fig. 12. X-gal signal in pancreas.**


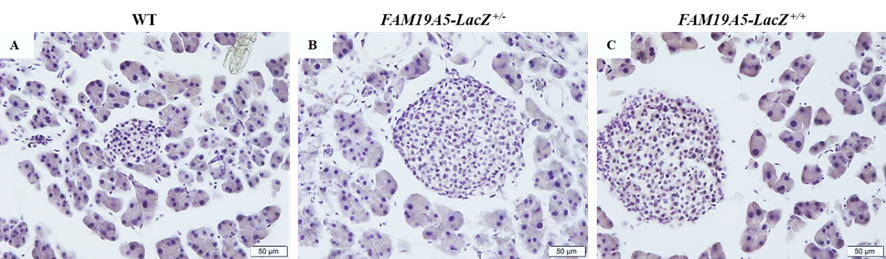


Representative light photomicrographs of pancreas cryosections of (A) wild type (WT, male #1), (B) *FAM19A5-LacZ*^+/-^ (heterozygote, male #1), and (C) *FAM19A5-LacZ*^+/+^ (homozygote, male #1) mice. The cryosections were stained with X-gal solution and counterstained with hematoxylin. No X-gal signals were detected in either WT or *FAM19A5-LacZ* KI mice.

**Supplementary Fig. 13. X-gal signal in liver.**


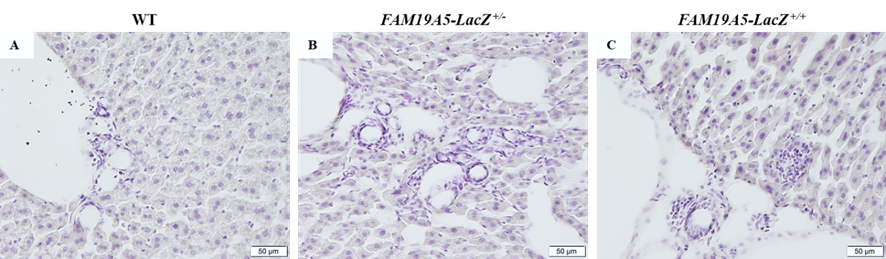


Representative light photomicrographs of liver cryosections of (A) wild type (WT, male #1), (B) *FAM19A5-LacZ*^+/-^ (heterozygote, male #1), and (C) *FAM19A5-LacZ*^+/+^ (homozygote, male #1) mice. The cryosections were stained with X-gal solution and counterstained with hematoxylin. No X-gal signals were detected in either WT or *FAM19A5-LacZ* KI mice.

**Supplementary Fig. 14. X-gal signal in salivary glands.**


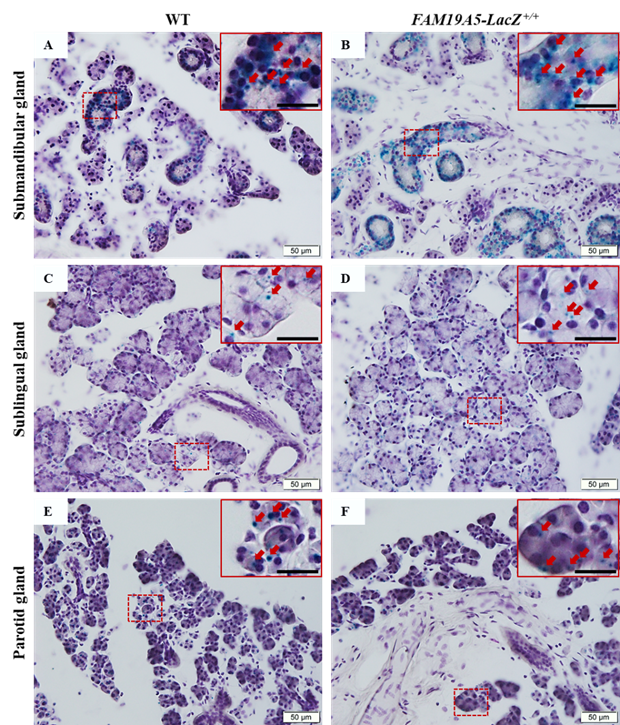


Representative light photomicrographs of salivary gland cryosections of (A, C, E) wild type (WT, male #2) and (B, D, F) *FAM19A5-LacZ*^+/+^ (homozygote, male #2 and #3) mice. The cryosections were stained with X-gal solution and counterstained with hematoxylin. The image in the dashed box is magnified in the inset, which is outlined in the same color as the dashed box. (A-B) Images showing the submandibular glands of WT male #2 and *FAM19A5-LacZ*^+/+^ male #2. Red arrows indicate punctate/dispersed blue precipitates in mucous acini observed in both WT and *FAM19A5-LacZ*^+/+^ mice. (C-D) These images show the sublingual glands of WT male #2 and *FAM19A5-LacZ*^+/+^ male #3. Red arrows indicate punctate blue precipitates in mucous acini observed in both WT and *FAM19A5-LacZ*^+/+^ mice. (E-F) These images show the parotid glands of WT male #1 and *FAM19A5-LacZ*^+/+^ male #2. Red arrows indicate punctate blue precipitates in serous acini observed in both WT and *FAM19A5-LacZ*^+/+^ mice. The scale bars in the inset represent 20 μm.

**Supplementary Fig. 15. X-gal signal in thymus.**


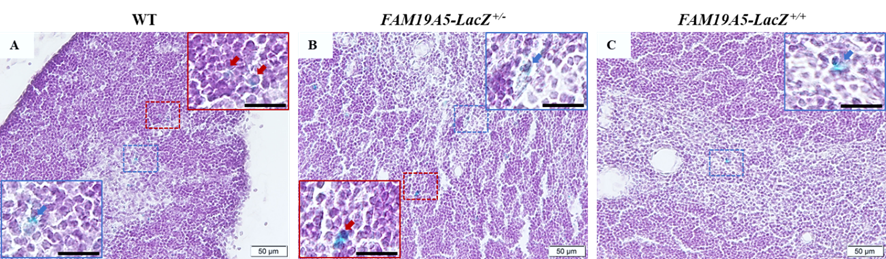


Representative light photomicrographs of thymus cryosections of (A) wild type (WT, male #1), (B) *FAM19A5-LacZ*^+/-^ (heterozygote, male #1), and (C) *FAM19A5-LacZ*^+/+^ (homozygote, male #1) mice. The cryosections were stained with X-gal solution and counterstained with hematoxylin. The image in the dashed box is magnified in the inset, which is outlined in the same color as the dashed box. Red arrows indicate dispersed blue precipitates in thymic cortex observed in both WT and *FAM19A5-LacZ* KI mice. The blue arrows indicate dispersed blue precipitates in thymic medulla observed in both WT and *FAM19A5-LacZ* KI mice. The scale bars in the inset represent 20 μm.

**Supplementary Fig. 16. X-gal signal in spleen.**


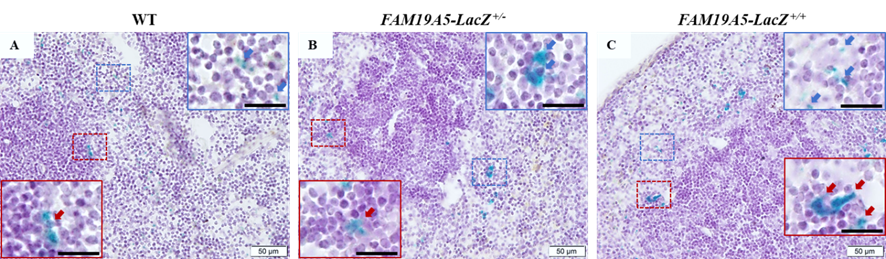


Representative light photomicrographs of spleen cryosections of (A) wild type (WT, male #1), (B) *FAM19A5-LacZ*^+/-^ (heterozygote, male #1), and (C) *FAM19A5-LacZ*^+/+^ (homozygote, male #1) mice. The cryosections were stained with X-gal solution and counterstained with hematoxylin. The image in the dashed box is magnified in the inset, which is outlined in the same color as the dashed box. Red arrows indicate dispersed blue precipitates in white pulp observed in both WT and *FAM19A5-LacZ* KI mice. The blue arrows indicate dispersed blue precipitates in red pulp observed in both the WT and *FAM19A5-LacZ* KI mice. The scale bars in the inset represent 20 μm.

**Supplementary Fig. 17. X-gal signal in bone marrow.**


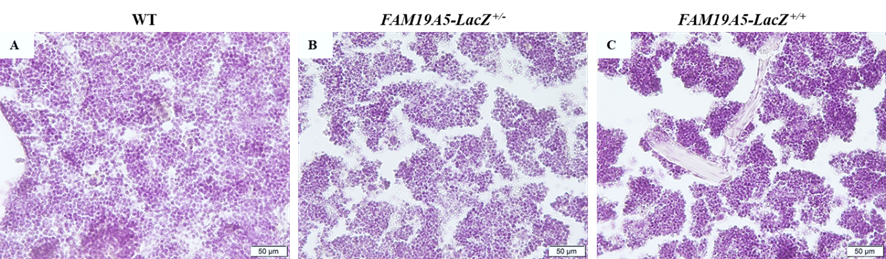


Representative light photomicrographs of femoral bone marrow cryosections of (A) wild type (WT, male #1), (B) *FAM19A5-LacZ*^+/-^ (heterozygote, male #1), and (C) *FAM19A5-LacZ*^+/+^ (homozygote, male #1) mice. The cryosections were stained with X-gal solution and counterstained with hematoxylin. No X-gal signals were detected in either WT or *FAM19A5-LacZ* KI mice.

**Supplementary Fig. 18. X-gal signal in pituitary gland.**


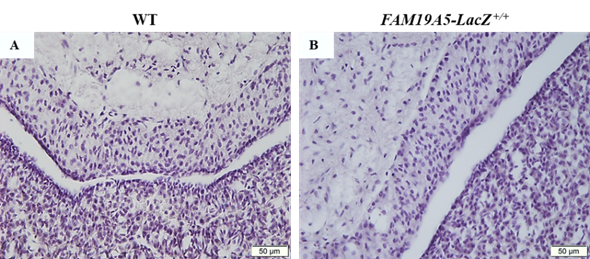


Representative light photomicrographs of pituitary gland cryosections of (A) wild type (WT, male #2) and (B) *FAM19A5-LacZ*^+/+^ (homozygote, male #2) mice. The cryosections were stained with X-gal solution and counterstained with hematoxylin. No X-gal signals were detected in either WT or *FAM19A5-LacZ*^+/+^ mice.

**Supplementary Fig. 19. X-gal signal in thyroid gland.**


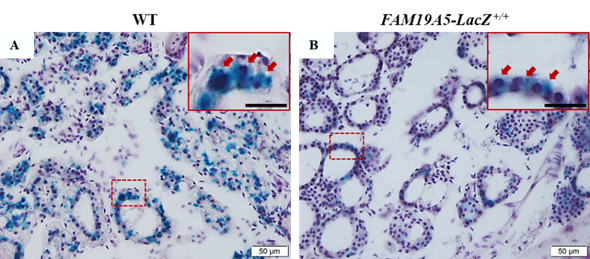


Representative light photomicrographs of thyroid gland cryosections of (A) wild type (WT, male #2) and (B) *FAM19A5-LacZ*^+/+^ (homozygote, male #3) mice. The cryosections were stained with X-gal solution and counterstained with hematoxylin. The image in the dashed box is magnified in the inset, which is outlined in the same color as the dashed box. Red arrows indicate dispersed blue precipitates in follicular cells observed in both WT and *FAM19A5-LacZ*^+/+^ mice. The scale bars in the inset represent 20 μm.

**Supplementary Fig. 20. X-gal signal in skeletal muscle.**


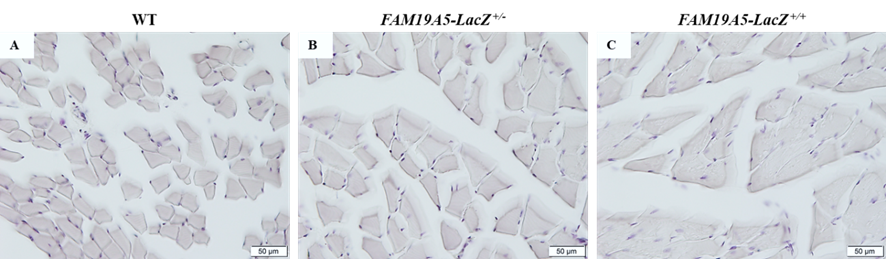


Representative light photomicrographs of femoral muscle cryosections of (A) wild type (WT, male #1), (B) *FAM19A5-LacZ*^+/-^ (heterozygote, male #1), and (C) *FAM19A5-LacZ*^+/+^ (homozygote, male #1) mice. The cryosections were stained with X-gal solution and counterstained with hematoxylin. No X-gal signals were detected in either WT or *FAM19A5-LacZ* KI mice.

**Supplementary Fig. 21. X-gal signal in white adipose tissue.**


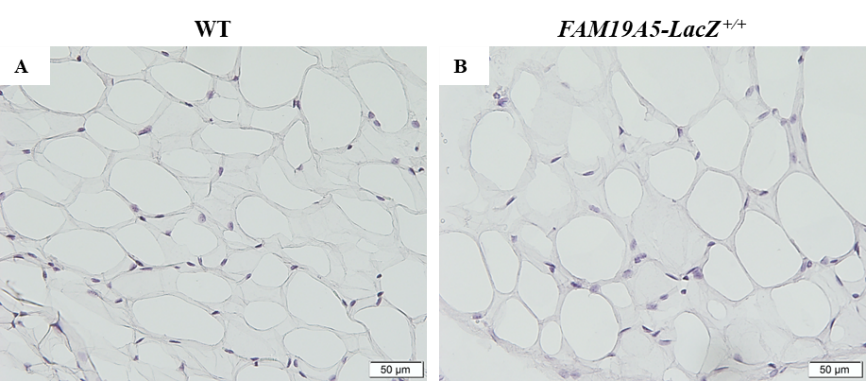


Representative light photomicrographs of white adipose tissue cryosections of (A) wild type (WT, male #1) and (B) *FAM19A5-LacZ*^+/+^ (homozygote, male #1) mice. All white adipose tissues were collected from the region adjacent to the reproductive system, epididymis or ovary. The cryosections were stained with X-gal solution and counterstained with hematoxylin. No X-gal signals were detected in either WT or *FAM19A5-LacZ*^+/+^ mice.

**Supplementary Fig. 22. X-gal signal in brown adipose tissue.**


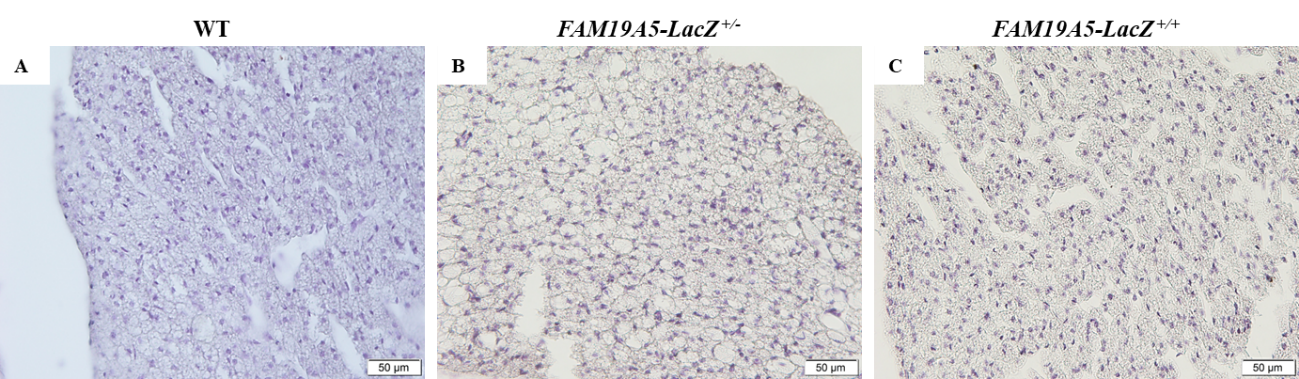


Representative light photomicrographs of brown adipose tissue cryosections of (A) wild type (WT, male #1), (B) *FAM19A5-LacZ*^+/-^ (heterozygote, male #1), and (C) *FAM19A5-LacZ*^+/+^ (homozygote, male #1) mice. All brown adipose tissues were collected from the region adjacent to the dorsal skin. The cryosections were stained with X-gal solution and counterstained with hematoxylin. No X-gal signals were detected in either WT or *FAM19A5-LacZ* KI mice.

**Supplementary Fig. 23. X-gal signal in skin.**


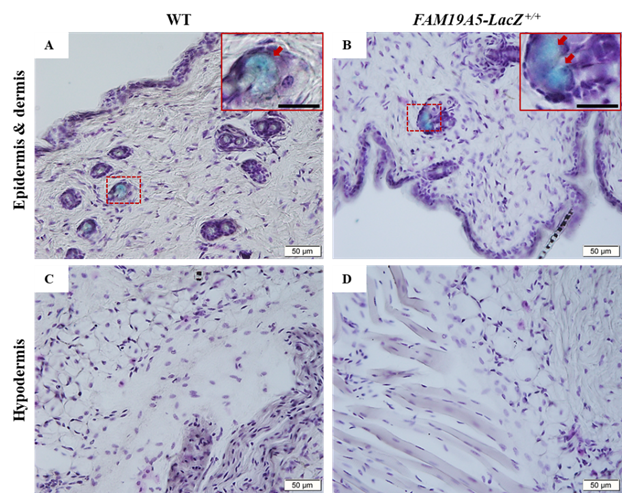


Representative light photomicrographs of skin cryosections of (A, C) wild-type (WT, male #2) and (B, D) *FAM19A5-LacZ*^+/+^ (homozygote, male #3) mice. All skin samples were collected from the parietal/occipital bone region. The cryosections were stained with X-gal solution and counterstained with hematoxylin. (A-B) These images show the epidermis and dermis regions of the skin. The image in the dashed box is magnified in the inset, which is outlined in the same color as the dashed box. Red arrows indicate dispersed blue precipitates in sebaceous glands observed in both WT and *FAM19A5-LacZ*^+/+^ mice. (C-D) These images show the hypodermis regions of the skin. No X-gal signals were detected in either the WT or *FAM19A5-LacZ*^+/+^ mice. The scale bars in the inset represent 20 μm.

**Supplementary Fig. 24. X-gal signal in seminal vesicle and coagulating gland.**


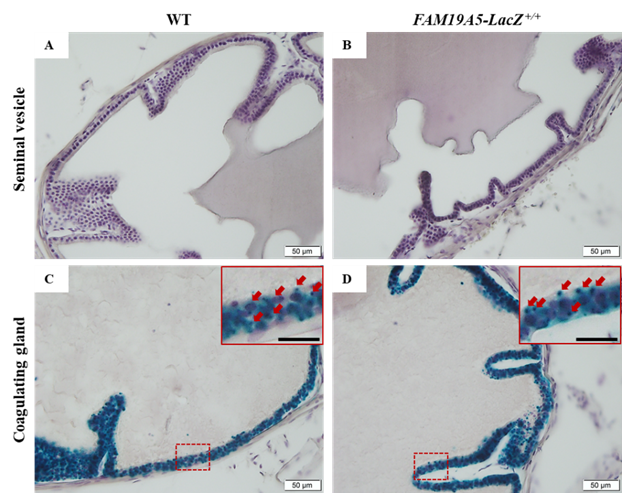


Representative light photomicrographs of seminal vesicles or coagulating gland cryosections of (A, C) wild-type (WT, male #2) and (B, D) *FAM19A5-LacZ*^+/+^ (homozygote, male #3) mice. The cryosections were stained with X-gal solution and counterstained with hematoxylin. (A-B) These images show seminal vesicles. No X-gal signals were detected in either the WT or *FAM19A5-LacZ*^+/+^ mice. (C-D) These images show coagulating glands. The image in the dashed box is magnified in the inset, which is outlined in the same color as the dashed box. Red arrows indicate punctate blue precipitates in coagulating gland epithelia observed in both WT and *FAM19A5-LacZ*^+/+^ mice. The scale bars in the inset represent 20 μm.
